# Differential Phagocytic Properties of CD45^low^ Microglia and CD45^high^ Brain Mononuclear Phagocytes—Activation and Age-Related Effects

**DOI:** 10.3389/fimmu.2018.00405

**Published:** 2018-03-02

**Authors:** Srikant Rangaraju, Syed Ali Raza, Noel Xiang’An Li, Ranjita Betarbet, Eric B. Dammer, Duc Duong, James J. Lah, Nicholas T. Seyfried, Allan I. Levey

**Affiliations:** ^1^Department of Neurology, Emory University, Atlanta, GA, United States; ^2^Department of Chemistry, Emory University, Atlanta, GA, United States; ^3^Department of Biochemistry, Emory University, Atlanta, GA, United States

**Keywords:** CNS mononuclear phagocytes, phagocytosis, flow cytometry, neuroinflammation, Alzheimer’s disease

## Abstract

In the central nervous system (CNS), microglia are innate immune mononuclear phagocytes (CNS MPs) that can phagocytose infectious particles, apoptotic cells, neurons, and pathological protein aggregates, such as Aβ in Alzheimer’s disease (AD). While CD11b^+^CD45^low^ microglia account for the majority of CNS MPs, a small population of CD11b^+^CD45^high^ CNS MPs is also recognized in AD that surround Aβ plaques. These transcriptionally and pathologically unique CD45^high^ cells have unclear origin and undefined phagocytic characteristics. We have comprehensively validated rapid flow cytometric assays of bulk-phase and amyloid β fibril (fAβ) phagocytosis and applied these to study acutely isolated CNS MPs. Using these methods, we provide novel insights into differential abilities of CD11b^+^ CD45^low^ and CD45^high^ CNS MPs to phagocytose macroparticles and fAβ under normal, acute, and chronic neuroinflammatory states. CD45^high^ CNS MPs also highly upregulate TREM2, CD11c, and several disease-associated microglia signature genes and have a higher phagocytic capacity for Aβ as compared to CD45^low^ microglia in the 5xFAD mouse model of AD that becomes more apparent with aging. Our data suggest an overall pro-phagocytic and protective role for CD11b^+^CD45^high^ CNS MPs in neurodegeneration, which if promoted, could be beneficial.

## Main Points

We demonstrate that CD11b^+^CD45^high^ cells, unlike CD45^low^ microglia, have a unique high capacity for phagocytosing amyloid β fibrils. In mouse models of AD pathology, CD45^high^ cells upregulate their ability to phagocytose amyloid β, highly express Trem2 and Cd11c, and more closely resemble disease-associated microglia that progressively emerge in AD.

## Introduction

Professional phagocytes, such as macrophages and microglia, in the brain internalize particles through pinocytosis, endocytosis, and phagocytosis using a diverse array of receptors. Phagocytosis typically involves engulfment of particles >0.5 μm in size and is an actin-dependent process unlike pinocytosis and endocytosis, which are clathrin-dependent (1). Phagocytic cells in the central nervous system (CNS) include microglia, macrophages (collectively called CNS mononuclear phagocytes or CNS MPs), as well as ependymal cells, pericytes, and astrocytes (2–4). Phagocytosis is a critical mechanism that facilitates uptake, degradation, and clearance of debris, infectious materials, and pathogenic protein aggregates seen in neurodegenerative diseases, such as Alzheimer’s disease (AD) (5). Genome-wide-association studies in humans have identified polymorphisms in CNS MP phagocytic genes, such as TREM2 and CD33, as independent risk factors for AD and preclinical studies in AD models further support key neuropathological roles for CNS MPs in neurodegeneration (6–8). In AD, dysregulation of phagocytic mechanisms in chronically activated microglia is associated with progressive amyloid β (Aβ) aggregation and pro-inflammatory neurotoxic responses (9).

Within the CNS, CD11b^+^ CD45^low^ microglia account for majority of CNS MPs while small proportions of CD11b^+^ CD45^high^ (<5%) cells are also present that may represent peripherally derived CNS-infiltrating macrophages or activated microglia that upregulate CD45 (10). These CD45^low^ and CD45^high^ subpopulations of CNS MPs have been found to have distinct transcriptomic profiles, and differential contributions of these subpopulations to neurodegenerative and neuroinflammatory diseases have been suggested (10–12). In AD mouse models, CD11b^+^ CD45^high^ CNS MPs express higher levels of TREM2, a phagocytic protein directly implicated in AD pathogenesis, suggesting Aβ-clearing roles for these cells (9, 13). However, their phagocytic activity is unknown, and a direct comparison of phagocytic capacity of CD11b^+^ CD45^low^ and CD11b^+^ CD45^high^ CNS MPs has not been performed.

As compared to peripheral tissue macrophages, microglia have lower phagocytic capacity which can be augmented following activation by pro-inflammatory stimuli (14, 15). Measurement of phagocytic capacity of microglia can be performed by *in vitro* engulfment assays using fluorescent probe-labeled particles, Aβ aggregates, opsonized bacteria, or latex particles followed by immunofluorescence microscopy (14, 16, 17). These assays can introduce sampling biases by exclusion of non-adherent cells in a cell type-like BV2 that has significant proportions of live floating cells. The majority of primary microglial phagocytosis studies have also been performed in cells maintained in culture for days to weeks. Since a rapid loss of microglial transcriptomic signatures is observed after isolation from the brain and *in vitro* culture (18), phagocytic studies using acutely isolated cells are more likely to reflect the complex CNS microenvironment in which microglia reside (19). Flow cytometric assays can have significant advantages, including large sampling fractions, higher sensitivity, the ability to rapidly phenotype phagocytic properties of freshly isolated microglia and macrophages, as well as to compare differences in phagocytic properties between subpopulations of cells within the same sample (20).

We report the validation of rapid flow cytometric assays of macroparticle and fibrillar Aβ_42_ (fAβ42) phagocytosis, each regulated by distinct phagocytic receptors. By applying these assays to study acutely isolated CNS MPs, we provide novel insights into differences in phagocytic properties of CD11b^+^ CD45^low^ and CD11b^+^ CD45^high^ CNS MPs under normal, acute, and chronic neuroinflammatory states. Based on our results, including expression patterns of pro-phagocytic proteins TREM2 and CD11c, transcriptomic profiling of CD11b^+^ CNS MPs, and profiling of their phagocytic properties, we conclude that CD11b^+^CD45^high^ cells are highly phagocytic CNS MPs with high affinity for Aβ in AD.

## Materials and Methods

### Reagents

Lipopolysaccharide (LPS) was purchased from Sigma-Aldrich (Cat # L4391, *E. coli* 0111:B4) and was used at a concentration of 100 ng/ml for *in vitro* experiments involving BV2 microglia. Dose of LPS used for intraperitoneal injections of mice was 20 μg/dose. Polystyrene red-fluorescent 1 µm microspheres (Thermo-Fisher Fluorospheres Cat #F13083) were used for microsphere phagocytosis experiments. Cells were exposed to 1 µl of microspheres (≈ microsphere to cell ratio > 100) for 30 min at 37°C, followed by washing and flow cytometry. Fluorophore-conjugated monoclonal antibodies for flow cytometry were purchased from BD Biosciences [anti-CD11b (APC-Cy7) and anti-CD45 (PE-Cy7)] and used according to the manufacturer’s instructions. HiLyte-488™ conjugated Aβ42 (Fluor 488 labeled, Eurogentec, Cat #AS-60479) monomeric solutions were prepared as described below. Unconjugated Aβ42 was incubated with HiLyte-488-Aβ42 (ratio 3:1) to yield fibrillar fAβ42-HiLyte [488] conjugates. Percoll for CNS MP isolation (#P1644) and Cytochalasin D (CytoD) (50 µM, #2618) were obtained from Sigma-Aldrich.

### Synthesis and Purification of Aβ42

Aβ42 was synthesized on a CEM Liberty peptide synthesizer. Fmoc-Ala-PAL-PEG-PS resin (Applied Biosystems) was swollen in 50% dimethylformamide (DMF)/50% dichloromethane for at least 15 min before the first deprotection reaction. Deprotections were performed with 20% v/v piperidine + 0.1 M hydroxybenzotriazole (HOBt) in DMF at 75°C for 3 min and couplings were performed with 1 M HOBt in DMF and 0.5 M N,N′-diisopropylcarbodiimide in DMF at 75°C for 5.5 min. However, histidine was double coupled at 50°C for 8 min and arginine was double coupled at 75°C for 10 min. The peptide was cleaved off the dried resin with 10 ml of trifluoroacetic acid (TFA)/thioanisole/1,2-ethanedithiol/anisole (90:5:3:2% v/v/v/v) at room temperature for 3 h. The mixture was then filtered drop-wise into cold (−20°C) diethyl ether and the precipitated peptide was centrifuged at 4,000 rpm, 4°C for 10 min. The pellet was washed thrice with cold diethyl ether by resuspension and centrifugation, then dried overnight in a desiccator. The crude peptide was then dissolved in 10 ml of 15% MeCN + 0.1% TFA and purified by RP-HPLC using a C18 column (Jasco) with a 1%/min MeCN-water + 0.1% TFA gradient. The eluates were collected in fractions and analyzed by MALDI-MS with α-cyano-4-hydroxycinnamic acid (Sigma-Aldrich) as the matrix. Fractions containing Aβ42 were pooled and acetonitrile was removed by rotary evaporation. The peptides were then lyophilized to dryness and the peptide content of the lyophilized powder was determined with the bicinchoninic acid (BCA) assay (Thermo Fischer Scientific) to correct for the mass of residual salts and water of hydration.

### Aβ42 Peptide Assembly

Aβ42 was pretreated with 10% w/v NH_4_OH and re-lyophilized to reduce the population of preexisting aggregates (21). The treated sample was then reconstituted to 150 µM by adding 1 mM NaOH to 90% of the final volume, bath-sonicated for 10 min, before the 10× buffer (100 mM sodium phosphate, pH 7.1) was added, bringing the final pH to 7.4. Then, 410.64 µl of that aliquot was added to 0.1 mg of HiLyte™ Fluor 488-Aβ42, to obtain a 200 µM total Aβ42 solution, of which 25% of the peptides are labeled. The sample was then incubated in the dark at room temperature for 7 days and then transferred to 4°C. The final concentrations of the conjugate fAβ42-HiLyte Alexa-488-labeled used for *in vivo* experiments with BV2 microglia were 0.5, 1, and 10 µM.

### Transmission Electron Microscopy (TEM)

Peptide solutions were incubated on TEM grids (200 mesh formvar/carbon-coated copper grids, Electron Microscopy Sciences) for 1.5 min. The excess solution was blotted away with filter paper, before the grid was stained with 2% w/v uranyl acetate in water for 1.5 min. Grids were imaged on a Hitachi HT7700 with a tungsten filament at an accelerating voltage of 80 kV and the pixel input ranges of the image files were adjusted in GIMP 2 (GNU Image Manipulation Program) for clarity.

### Animals

C47BL/6J mice (JAX 000664) were housed in the Department of Animal Resources at Emory University under standard conditions with no special food/water accommodations. Institutional Animal Care and Use Committee approval was obtained prior to *in vivo* work and all work was performed in strict accordance with the Guide for the Care and Use of Laboratory Animals of the National Institutes of Health. To induce acute neuroinflammation, adult mice were given intraperitoneal LPS injections (10 µg/dose × 4 daily doses) after which a sickness response is typically observed (22). If weight loss was ≥25%, animals were euthanized.

### Primary Microglial Isolation

Adult C57BL6 mice (16–24 weeks) were euthanized, and brains were obtained after rapid cold saline cardiac perfusion and CNS MPs were isolated as previously described (23). Brains were minced over a 40-µm cell strainer, and single-cell suspensions were washed in PBS in a centrifuge for 5 min at 800 × *g* at room temperature. Pellets were resuspended in 5 ml of Dulbecco’s Modified Eagle Medium (DMEM), spun at 1,200 × *g* for 5 min and supernatants removed. Cell pellets were then resuspended in 6 ml per brain of 37% stock isotonic percoll (SIP) solution (90% percoll + 10% 10xHBSS). The cell suspension was transferred into 15 ml conical tubes and 2 ml of 70% SIP was slowly under laid. Then on top of the 37% layer, 2 ml of 30% SIP was slowly layered. The gradient established was then centrifuged for 30 min at 800 × *g* without deceleration at 20°C. The top layer of floating myelin was then removed and a Pasteur pipette was used to collect 2 ml from 70 to 37% interphase without disturbing the 70% layer into a clean 15 ml tube containing 10 ml cold PBS and washed three times. The pellets comprised of CNS MPs were resuspended in 500 µl of PBS or warm DMEM containing 10% fetal bovine serum. The cells were then used for *in vitro* phagocytosis assays and flow cytometry as described below. In some experiments, splenocytes were isolated after the spleen was homogenized through a cell strainer, followed by RBC lysis. The pellets of splenocytes were then used for phagocytosis and flow cytometric studies.

### BV2 Cell Culture

BV2 cells were cultured in DMEM supplemented with 10% FBS and penicillin and passaged every 3–4 days after reaching confluence. Cells were discarded after 20 passages due to observed morphological and functional alterations.

### Fluorescent Polystyrene Microsphere Phagocytosis Assay

The size, complexity, and fluorescence profiles of microspheres alone were first determined by flow cytometry. Phycoerythrin (PE)-conjugated polystyrene microspheres (2 µl/tube, ≈200 microspheres/microglial cell) were added to BV2 cells or freshly isolated CNS MPs at 37°C in a 5% CO_2_ humidified incubator after which flow cytometry was performed. For BV2 microglia, no additional label was used. In a series of experiments, BV2 cells exposed to microspheres were sorted based on degree of fluorescence observed. Since a unitary pattern of phagocytosis was observed with microspheres, we were able to easily sort out cells at various peaks of fluorescence. These sorted cells were then allowed to adhere to poly-l-lysine coated coverslips and then labeled with CD45-FITC mAb for 30 min and then imaged on an immunofluorescence microscope. This allowed us to correlate fluorescence peaks observed by flow cytometry with number of beads phagocytosed by each cell.

Concentrations of microspheres (0.5–2 µl/tube) and incubation times were varied to obtain optimal labeling and microsphere uptake. We performed optimization experiments to determine the minimum duration of incubation (time duration ranging between 15 min and 1 h) that resulted in near-maximal phagocytic uptake of PE-microspheres by BV2 cells. In some experiments, BV2 cells were pretreated with CytoD (50 µM) for 30 min at 37°C to specifically inhibit actin-dependent phagocytosis and then thoroughly washed. The effect of LPS (100 ng/ml × 24 h) on phagocytosis was also determined *in vitro* as well as *in vivo* (intraperitoneal LPS 10 µg/dose/mouse × 4 daily doses). Cells were incubated with microspheres for 30 min after which 1 ml of cold PBS was added and the cells were harvested by gentle pipetting (4–5 times). Greater than 95% cell viability using this method was confirmed using a flow cytometric marker of non-viability (eBioscience eFluor 506, #65-0866-14) as well as optimal gating of live cells using forward and side scatter (SSC) profiles. The cells were finally collected in 5 ml polystyrene flow cytometry compatible tubes. The assay using BV2 cells was also optimized for use in a 96-well plate format. For CNS MPs, freshly isolated cells were first exposed to microspheres for 30 min at 37°C, washed with cold PBS, and then labeled with fluorophore-conjugated CD11b (CD11b-APC-Cy7, BD Biosciences #557657) and CD45 (CD45-FITC, BD Biosciences #553080) antibodies for 30 min at 4°C followed by washing prior to flow cytometry (24, 25).

### Fluorescent Fibrillar Aβ42 Phagocytosis Assay

Fibrillar fluorescent fAβ42 (conjugated to HiLyte Fluor 488) was prepared as described above. HiLyte monomers (Aβ42, HiLyte™ Fluor 488-labeled) were prepared by mixing 100 µg of peptide in 50 µl of dimethyl sulfoxide to linearize the peptide and then diluted in PBS to 1 ml solution to obtain a final stock solution of 20 µM which was used within 30 min for our studies since intermediate levels of aggregation occur by 1 h of dilution. Phagocytosis of fAβ42-488 (0.5–10 µM concentrations) with varying time intervals (30, 60, and 120 min) and of Aβ42-488 monomer (2.5 µM, equivalent to 10 µM fAβ42-488) was performed by incubating BV2 cells with either of the reagents at 37°C. The steps following Aβ incubation were similar to those for the microsphere assay. Pre-incubation with CytoD (50 µM) was also performed in separate experiments to determine the proportion of fAβ42 uptake that was actin-dependent (26).

### Functional Blockade of Phagocytosis

Functional blockade studies of phagocytosis of both PE-microspheres and fAβ42 were performed using previously confirmed neutralizing antibodies against TLR2, CD148/PTPRJ, CD36, and MSR1 (27–31). Blocking antibodies against TLR2 (1:50, stock 0.1 mg/ml; Cat# mabg-mtlr2) (30, 31), CD148/PTPRJ (1:100, stock 1 mg/ml; Cat# MABC87) (28), MARCO (1:250, stock 100 µg/ml, Cat# HM1068) (32), CD36 (1:250, stock 1 mg/ml, Cat# ab23680) (29), and MSR1 1:10, stock 0.2 mg/ml; Cat# 1797-MS (27) receptors were used in our experiments. BV2 cells were first incubated with the above blockers for 1 h after which either PE-microspheres or fAβ42 were added for 30 min followed by washing and flow cytometric studies as described above.

### Flow Cytometric Studies and Fluorescence Activated Cell Sorting (FACS)

First, the flow cytometric properties of PE-microspheres alone were characterized. Median fluorescence intensity (MFI) was used to quantify fluorescence of each observed discrete peak: first peak corresponding to the complexity of a single microsphere, peak 2 corresponding to twice the complexity of a single microsphere and so on. However, all the particles corresponded to the same flow cytometric measure of size, confirming that all microspheres were of a uniform size. The observed MFI for each peak was plotted against estimated MFIs using the MFI for first peak and a correlation coefficient determined. BV2 cells were harvested and washed after incubation with microspheres or Aβ_42_. CNS MPs were immuno-labeled with anti-CD11b and anti-CD45 fluorophore-conjugated mAbs. Compensation was performed prior to each experiment using single fluorophore labeled compensation beads. Sample data acquisition was always preceded by running an unstained cell sample (without microspheres or fAβ42) after which a positive control was run at low speed to allow appropriate voltage adjustment especially for PE due to very bright fluorescence of these microspheres. Aβ42 fluorescence was acquired in the Alexa-488 channel. Microsphere and Aβ42 phagocytosis experiments were performed independently of each other. BV2 cells were gated based on FSC and SSC characteristics that identify viable cells (typically 70–80% of all isolated cells). For studies with CNS MPs, we first gated for live cells using FSC/SSC gating, followed by CD11b and CD45 gating to identify CD11b^+^ CD45^low^ and CD11b^+^ CD45^high^ populations after which phagocytosis within each subset was assessed. All flow cytometric data were analyzed using FlowJo version 10. For microsphere phagocytosis studies, distinct peaks were identified in the cell distribution while for Aβ42 phagocytosis, a shift in fluorescence and a second peak of fluorescent cells were observed. CytoD pre-incubation experiments allowed us to confirm actin-dependence of these observed responses. For cell sorting, washed BV2 cells were subjected to FACS using FACSAria IIB instrument as per standard sorting protocols. Purity of sorted populations was immediately assessed after the sort before further experimentation.

### Immunofluorescence Microscopy

BV2 cells sorted based on observed peaks [No staining, Peak 1, Peak 2, Peak 3, and Higher (Peak > 4)] after incubation with PE-microspheres were plated on poly-l-Lysine-coated glass coverslips and 2 ml of DMEM was then added to each well followed by incubation for 1 h to allow adherence. Medium was gently aspirated and the cells fixed using 2% paraformaldehyde for 15 min on ice. Cells were then incubated with Hoescht nuclear dye (1:10,000) and anti-CD45-FITC (1:100) for 30 min on ice. Cells were then washed followed by image acquisition on an immunofluorescence microscope (Microscope: Olympus BX51 and camera: Olympus DP70). Imaging processing and image overlay was performed using ImageJ.

### Mass Spectrometry Sample Preparation

For *in vitro* stimulation of BV2 microglia, we cultured BV2 cells with either LPS (100 ng/ml), recombinant mouse IL4 (20 ng/ml, R&D Systems), or a combination of recombinant mouse IL10 (20 ng/ml, R&D Systems) and TGFβ1 (10 ng/ml, R&D Systems) for 48 h to induce M1-like and M2-like polarized activation states, after which cells were harvested by scraping followed by three washes in cold PBS (33). BV2 microglia samples were prepared essentially as described (23, 34). Each cell pellet was individually homogenized in 300 µl of urea lysis buffer (8 M urea, 100 mM NaHPO_4_, pH 8.5), including 3 µl (100× stock) HALT protease and phosphatase inhibitor cocktail (Pierce). All homogenization was performed using a Bullet Blender (Next Advance) according to manufacturer protocols and as previously published (34). Briefly, each cell pellet was added to Urea lysis buffer in a 1.5-ml Rino tube (Next Advance) harboring 750-mg stainless steel beads (0.9–2 mm in diameter) and blended twice for 5 min intervals in the cold room (4°C). Protein supernatants were transferred to 1.5 ml Eppendorf tubes and sonicated (Sonic Dismembrator, Fisher Scientific) three times for 5 s with 15 s intervals of rest at 30% amplitude to disrupt nucleic acids and subsequently vortexed. Protein concentration was determined by the BCA method, and samples were frozen in aliquots at −80°C. Protein homogenates (100 µg) were diluted with 50 mM NH_4_HCO_3_ to a final concentration of less than 2 M urea and then treated with 1 mM dithiothreitol at 25°C for 30 min, followed by 5 mM iodoacetamide at 25°C for 30 min in the dark. Protein was digested with 1:100 (w/w) lysyl endopeptidase (Wako) at 25°C for 2 h and further digested overnight with 1:50 (w/w) trypsin (Promega) at 25°C. Resulting peptides were desalted with a Sep-Pak C18 column (Waters) and dried under vacuum. For LC-MS/MS analysis, derived peptides were resuspended in 100 µl of loading buffer (0.1% formic acid, 0.03% TFA, 1% acetonitrile). Peptide mixtures (2 µl) were separated on a self-packed C18 (1.9 µm, Dr. Maisch, Germany) fused silica column [25 cm × 75 µM internal diameter (ID); New Objective, Woburn, MA, USA] by a Dionex Ultimate 3000 RSLCNano and monitored on a Fusion mass spectrometer (Thermo-Fisher Scientific, San Jose, CA, USA). Elution was performed over a 2 h gradient at a rate of 400 nl/min with buffer B ranging from 3 to 80% (buffer A: 0.1% formic acid in water, buffer B: 0.1% formic acid in acetonitrile). The mass spectrometer cycle was programmed to collect at the top speed for 3-s cycles. The MS scans (400–1,600 *m/z* range, 200,000 AGC, 50 ms maximum ion time) were collected at a resolution of 120,000 at *m/z* 200 in profile mode and the HCD MS/MS spectra (0.7 *m/z* isolation width, 30% collision energy, 10,000 AGC target, 35 ms maximum ion time) were detected in the ion trap. Dynamic exclusion was set to exclude previous sequenced precursor ions for 20 s within a 10 ppm window. Precursor ions with +1, and +8 or higher charge states were excluded from sequencing.

### MaxQuant for Label-free Quantification (LFQ) and Data Analysis

Raw data files were analyzed using MaxQuant v1.5.2.8 with Thermo Foundation 2.0 for RAW file reading capability, as previously published (23, 34). The search engine Andromeda was used to build and search a concatenated target-decoy IPI/Uniprot mouse reference (downloaded August 14, 2015). Protein Methionine oxidation (+15.9949 Da), and protein N-terminal acetylation (+42.0106 Da) were variable modifications (up to five allowed per peptide); cysteine was assigned a fixed carbamidomethyl modification (+57.0215 Da). Only fully tryptic peptides were considered with up to two mis-cleavages in the database search. A precursor mass tolerance of ±10 ppm was applied prior to mass accuracy calibration and ±4.5 ppm after internal MaxQuant calibration. Other search settings included a maximum peptide mass of 6,000 Da, a minimum peptide length of six residues, and 0.6 Da Tolerance for ion-trap HCD MS/MS scans. Co-fragmented peptide search was enabled to deconvolute multiplex spectra. The false discovery rate for peptide spectral matches, proteins, and site decoy fraction were all set to 1%. Quantification settings were as follows: re-quantify with a second peak finding attempt after protein identification has completed; match full MS1 peaks between runs; a 1.5-min retention time match window was used after an alignment function was found with a 20-min RT search space. The label-free quantification (LFQ) algorithm in MaxQuant (35, 36) was used for protein quantitation. Proteins with more than 25 percent overall missing data or more than 1 missing data point per treatment group were excluded from analysis.

### Secondary Analyses of Existing Mouse CNS MP RNASeq Data

RNAseq expression data files were downloaded from two sources: (1) NCBI Bioproject #PRJNA307271 that included population-level data from CD11b^+^CD45^high^ and CD45^low^ CNS MPs derived from adult WT mice as well as from WT mice injected with LPS to induce neuroinflammation (12), and (2) Supplemental data from a single-cell RNAseq study of CD45^+^ CNS MPs from WT and 5xFAD mice (37). From the single-cell RNAseq study, disease-associated microglia (DAM) genes and homeostatic microglial genes were identified as those with at least twofold higher expression as compared to the other group pairwise *t*-test *p* value < 0.01. From the other study, genes differentially expressed by LPS treatment were defined as those with at least twofold change in either direction. Genes highly expressed by CD11b^+^ CD45^high^ CNS MPs as compared to CD11b^+^ CD45^low^ microglia were identified based on relative expression (log2-fold change). These log2-transformed relative expression data were merged based on gene symbol and then used for analysis using Morpheus (Broad Institute) for hierarchical cluster analysis using the average linkage method (one minus Pearson correlation) and for heat map generation.

### Statistical Considerations

Graph-Pad version 5.0 was used to create all the graphs. Data are shown as mean ± standard error of the mean (SEM). *t*-Test (two tailed, assuming equal variance) was used for pairwise comparisons, with statistical significance set at *p* ≤ 0.05 unless otherwise specified. FlowJo version 10 was used for flow cytometry data analyses and profile generation in the layout editor. Microsoft Excel (version 2010) was used for data entry and correlation analyses.

## Results

### Validation of Flow Cytometric Assays of Fluorescent Polystyrene Microsphere and fAβ_42_ Phagocytosis in BV2 Microglia

**Figure 1:**
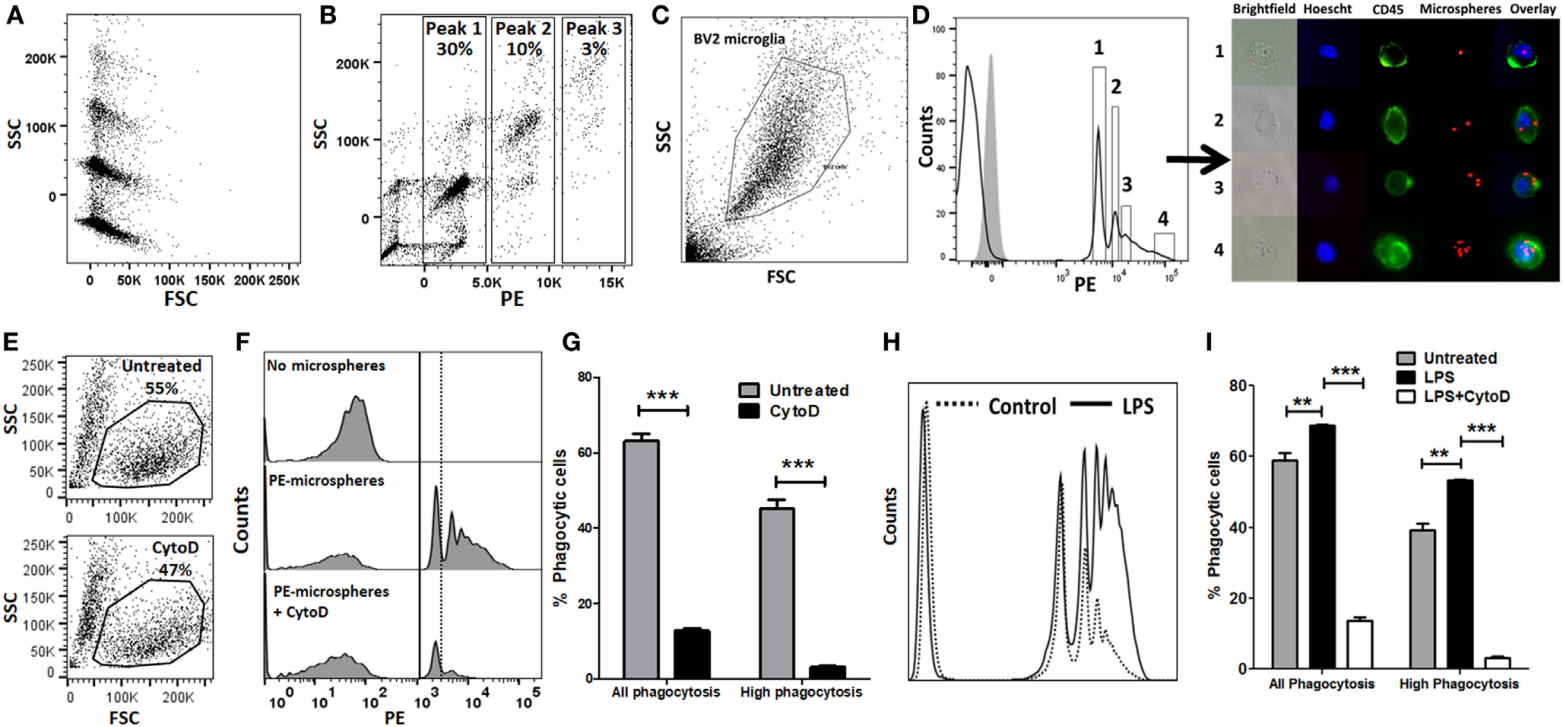
Validation of flow cytometric phycoerythrin (PE)-microsphere assay in BV2 microglia. **(A)** Forward scatter (FSC) and side scatter (SSC) profile of microspheres. **(B)** PE fluorescence vs. SSC. **(C)** Live cell gating strategy of BV2 microglia based on FSC and SSC. **(D)** Profile of PE-microsphere phagocytosis in resting BV2 cells. Four populations were identified for cell sorting. Gray histogram is fluorescence observed in untreated BV2 cells not exposed to microspheres. Immunofluorescence microscopy imaging of live fluorescence activated cell sorting-sorted BV2 cells. Blue: Hoescht nuclear stain, green: CD45-FITC, red: PE-microspheres. **(E)** Effect of CytoD treatment on overall cell viability. **(F,G)** Representative flow cytometric histograms and analyses comparing PE-microsphere phagocytosis with or without 50 µM CytoD pre-incubation. **(H,I)** Comparison of proportions of PE-microsphere phagocytosing cells after lipopolysaccharide (LPS) stimulation (100 ng/ml) for 24 h and inhibition by CytoD (50 µM). CytoD was added to BV2 cells after LPS stimulation but 30 min prior to addition of fAβ42-488 (10 µM).

We performed *in vitro* experiments to validate flow cytometric assays of phagocytosis using BV2 microglia as a model system. PE-conjugated 1 µm polystyrene microspheres were used to study bulk-phase phagocytosis of macroparticles while fAβ42 conjugated to Hilyte488 was used to study phagocytosis of protein aggregates to model AD. We first determined the fluorescence and flow cytometric characteristics of 1 µm PE-conjugated polystyrene microspheres. All microspheres had similar forward scatter characteristics indicating very similar sizes, however heterogeneity in SSC profiles (particle complexity) and degree of PE labelling were observed (Figures 1A,B). Distinct PE-labeled microsphere population peaks (peaks 1, 2, and 3 in Figure 1B) are representative of integral multiples of complexity and fluorescence of single microspheres. The majority of PE-labeled microspheres had similar forward and SSC characteristics, were single particles rather than clumps, and had predominantly uniform PE fluorescence intensities. We then incubated BV2 microglia with PE-microspheres and found that 20–30% of all BV2 cells took up at least 1 fluorescent bead by 30 min. In our optimization experiments, we observed maximal PE-microsphere uptake with 2 µl microspheres/reaction and 30 min incubation periods. 1 h and 30 min exposure resulted in equal microsphere phagocytosis while 15 min incubation resulted only in <10% uptake. Among the cells that phagocytosed microspheres, a complex cell distribution with multiple fluorescence peaks was observed, with peak 1 being most frequent (Figures 1C,D). A large proportion of BV2 cells did not exhibit any fluorescence indicating lack of phagocytic uptake or uptake of non-fluorescent microspheres. We then confirmed that the MFI of peak 1 of pure microspheres coincided with the MFI of peak 1 from BV2 cells that were incubated with microspheres. Next, we sorted four populations of BV2 cells (Peak 1, Peak 2, Peak 3, and Peak > 4). Post-sort purity was adequate (peak 1: 76%, peak 2: 65%, peak 3: 85%, and peak 4: 98%). Cells were directly visualized individually by fluorescence microscopy, and cells isolated from peak 1 contained one microsphere, peak 2 corresponded to two microspheres while very high-level phagocytosis was seen in the last population (Figure 1D). CytoD, an actin polymerization inhibitor that inhibits phagocytosis (15, 26, 38–42), decreased all peaks of microsphere phagocytosis (Figures 1E–G). However, CytoD did not abolish all phagocytic activity and about 10–15% of cells were able to bind at least 1 bead suggesting some non-specific cell surface binding or actin-independent uptake. High-level phagocytosis (peak ≥2) decreased from 43.4 to 3.17% following CytoD pre-treatment. Based on these observed phagocytic profiles, we defined metrics of phagocytic capacity including proportions of all phagocytic cells (peak ≥ 1) and highly phagocytic cells (peak ≥ 2). Since activation by pro-inflammatory stimuli also alters phagocytic properties of macrophages and microglia, we determined the effect of pro-inflammatory stimulus LPS on phagocytic characteristics (16, 43–50). LPS (100 ng/ml × 24 h) significantly augmented phagocytic responses in the microsphere phagocytosis assay and CytoD strongly inhibited this response (Figures 1H,I).

**Figure 2:**
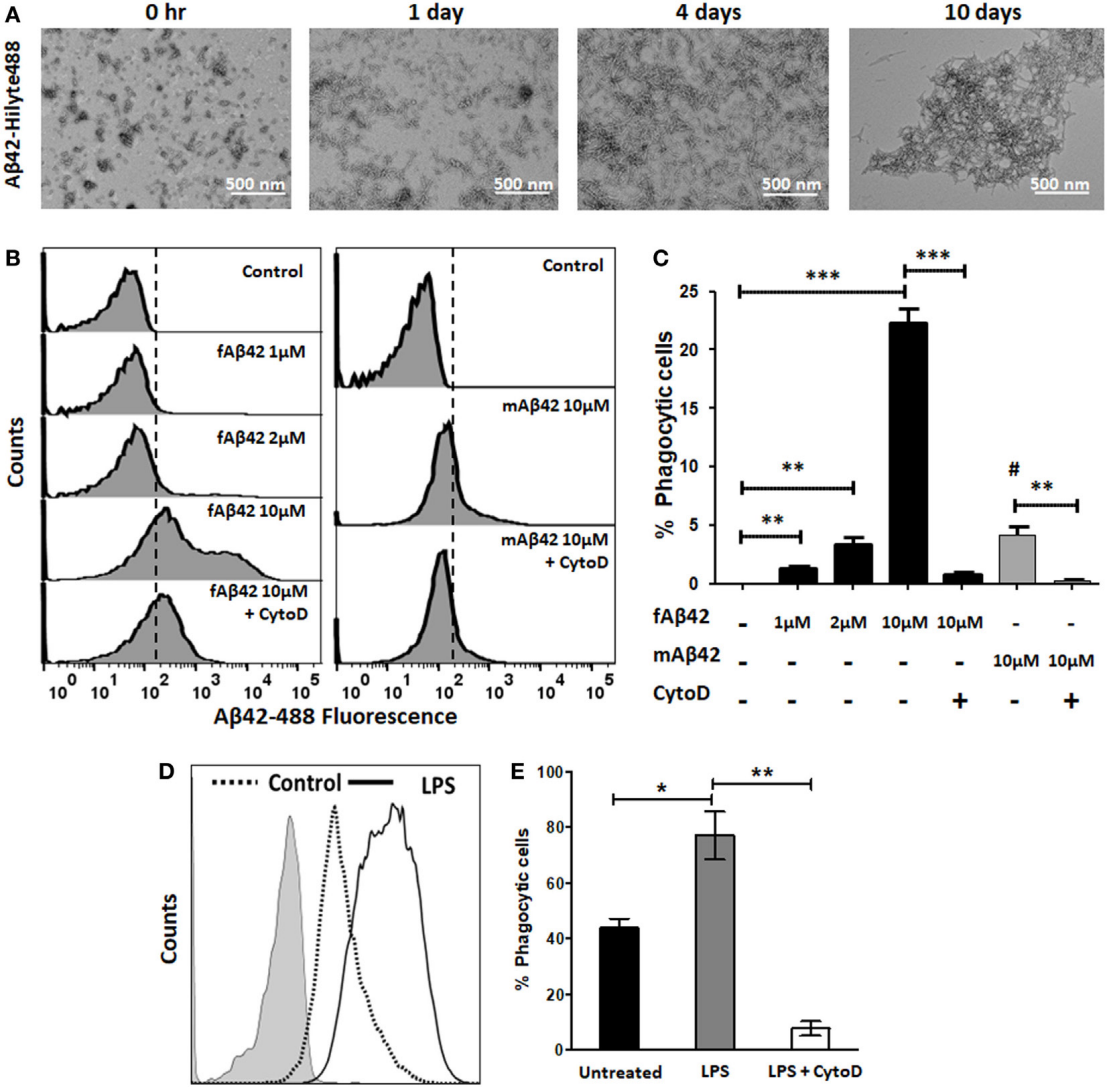
Validation of flow cytometric fluorescent fAβ42 phagocytosis assay in BV2 microglia. **(A)** TEM imaging showing Aβ42 fibril formation over a 10-day time course. **(B,C)** Representative flow cytometric histograms and quantitative analysis showing uptake of fAβ_42_-Hilyte488 by BV2 microglia and inhibition by Cyto D (Left); non-phagocytic binding of monomeric Aβ_42_-Hilyte488 shown on the right. Dotted line represents quantitation of data from five independent experiments. ^#^ in panel **(C)** represents statistical comparison of mAβ42 to the negative control (*p* = 0.01). **(D,E)** Comparison of proportions of fAβ42-phagocytosing cells after lipopolysaccharide (LPS) stimulation (100 ng/ml) for 24 h and inhibition by CytoD (50 µM). CytoD was added to BV2 cells after LPS stimulation but 30 min prior to addition of fAβ42-488 (10 µM). *N* = 5 independent experiments per condition (*p < 0.05, **p < 0.01, ***p < 0.005).

We then characterized the flow cytometric profile of fAβ42-Hilyte488 phagocytosis by BV2 microglia and used non-fibrillar HiLyte Aβ42-488 monomers as control (Figure 2). For Aβ42-Hilyte488 preparation, three parts of non-fluorescent Aβ_42_ and one part of Aβ42-Hilyte488 was used during fibril formation. TEM images of fAβ42-488 at different time points are shown (Figure 2A) and confirm robust fibril formation by day 4. Across the range of fAβ42 concentrations used (Figure 2B), we observed optimal uptake by unstimulated BV2 microglia at 10 µM concentration (equivalent to 2.5 µM of the monomeric Hilyte-488 component). In comparison, no shift in fluorescence was observed at lower concentrations (<1 μM fAβ42). A slight shift in fluorescence was observed with 2.5 µM monomeric fAβ42-488 which most likely represented non-specific membrane binding (Figure 2C). CytoD significantly inhibited fAβ42-488 phagocytosis and specifically abolished the second fluorescent peak without impacting the first peak (Figures 2B,C). Based on these results, we defined fAβ42 phagocytosis using the second fluorescence peak to indicate cells that had phagocytosed fAβ42. Since CytoD or Aβ42 especially at high concentrations can be cytotoxic, we also confirmed that cell viability in the BV2 cells was not affected by 30 min exposure to 50 µM CytoD or 10 µM fAβ42 (data not shown). LPS also augmented fAβ42 phagocytosis while CytoD inhibited this response (Figures 2D,E). In summary, these results characterize and validate our flow cytometric assays of PE-microsphere and fluorescent fAβ42 phagocytosis in microglia.

### Distinct Phagocytic Receptor Profiles of Resting, Pro-inflammatory, and Anti-inflammatory Activated Microglial States

**Figure 3:**
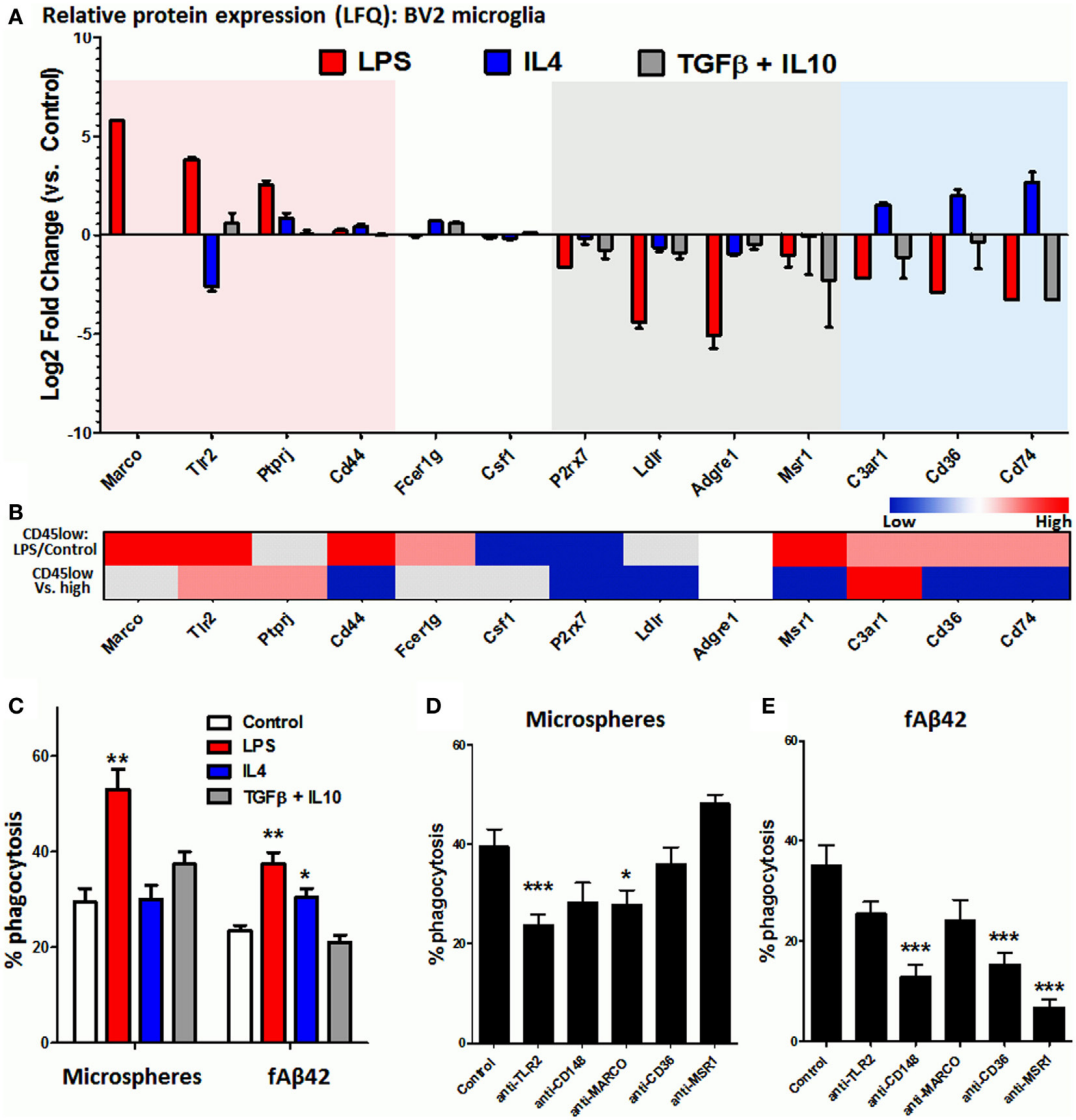
Identification of phagocytic receptors that regulate microsphere and fAb42 phagocytosis in BV2 microglia. **(A)** Differentially expressed phagocytic proteins identified by quantitative proteomics in BV2 microglia that were exposed to M1 [lipopolysaccharide (LPS)], M2a (IL4), and M2c (IL10 + TGFβ) polarizing stimuli for 48 h. Please refer to Table S1 in Supplementary Material for additional information. **(B)** Relative expression of BV2 phagocytic proteins at the transcriptomic level in acutely isolated CNS MPs, comparing LPS-treated vs. untreated CD45^low^ CNS MPs and CD45^low^ vs. CD45^high^ CD11b^+^ CNS MPs. Red indicates relative upregulation, Blue indicates relative downregulation and Gray indicates genes/proteins not identified by RNAseq. These data were obtained from publicly available RNAseq data. **(C)** Comparison of phycoerythrin (PE)-microsphere and fAβ42 phagocytic profiles of classically activated (LPS), alternatively activated (IL4), and deactivated (TGFβ + IL10) BV2 microglia. **(D,E)** Effects of functional blockade of selected phagocytic receptors on PE-microsphere **(D)** and fAβ42 phagocytosis **(E)** by BV2 microglia. *N* = 5 independent experiments; *p < 0.05, **p < 0.01, ***p < 0.005.

Microglia and CNS MPs adopt a diverse and complex array of activation states with distinct transcriptomic profiles that are now being revealed by RNA sequencing studies (12, 37, 51). *In vitro*, a polarized pro-inflammatory M1-like can be induced by LPS while anti-inflammatory and phagocytic M2-like profiles can be induced by cytokines including IL4, IL13, IL10, TGFβ, and corticosteroids (33, 47, 52, 53) although these *in vitro* states may only partially represent microglial activation states in the brain (54). Microglia, like peripheral macrophages, express a diverse array of phagocytic receptors including scavenger receptors (MARCO, MSR1/SR-A1), Fc receptors and associated proteins (FCGRs, CD148/PTPRJ), toll-like receptors (TLR2/4), and others including CD36 and TREM2 (15). In order to broadly determine the phagocytic receptor profile of BV2 microglia in resting and activated states, we performed label-free quantitative proteomics using whole cell lysates of BV2 cells exposed to *in vitro* polarizing stimuli including LPS to induce an M1-like state, and IL4 or combination of TGFβ + IL10 to induce M2-like polarized states (33). Within the 4,724 proteins identified by mass spectroscopy using LFQ (Table S1 in Supplementary Material), we searched for phagocytic proteins that were differentially expressed across the experimental conditions. In the Gene Ontology (GO) database (55), we identified 433 GO IDs (97 mouse genes) with any of the following annotations: “microglia, macrophage, receptor, phagocyte, phagocytosis or endocytosis.” This list served as a reference list of phagocytic mouse genes for purposes of this study (Table S2 in Supplementary Material). Of these phagocytic genes, 28 were identified in our BV2 proteome and their patterns of expression are shown in Figure 3A. LPS-upregulated phagocytic proteins included MARCO, TLR2, and PTPRJ (CD148), while phagocytic proteins specific to the IL4-upregulated state included complement receptor C3AR1, CD36, and CD74. Homeostatic phagocytic proteins that were downregulated regardless of treatment condition included P2RX7, LDLR, ADGRE1, and MSR1, while CD44, FCER1G, and CSF1 showed no significant changes. TREM2 protein was not identified in our proteomic data. We did not find any deactivated state-specific phagocytic protein signature in this analysis.

Although BV2 microglia represents a model system to study microglial activation, several differences as compared to primary microglia also exist (56, 57). To compare phagocytic receptor expression in BV2 and primary microglia, we searched existing RNAseq data from CD11b^+^ CD45^low^ and CD11b^+^ CD45^high^ murine CNS MPs from adult wild-type mice (P60), including adult mice that were treated with LPS *in vivo* (12). Of the 96 phagocytic genes identified in this RNASeq dataset, all BV2 phagocytic receptors were identified except for ADGRE1 (Figure 3B). Congruency between LPS-induced changes observed in BV2 (Figure 3A) and in CD45^low^ microglia (Figure 3B, upper row) was investigated. Upregulation of TLR2, MARCO, and CD44 and downregulation of P2RX7 and CSF1 were consistent, while several disagreements were observed for PTPRJ, C3AR1, CD36, and CD74 (Figure 3B). In RNASeq data comparing CD45^low^ to CD45^high^ CNS MPs (Figure 3B, lower row), we observed that C3AR1 was most highly expressed in CD45^low^ microglia, followed by TLR2 and PTPRJ/CD148, while CD44, P2RX7, LDLR, MSR1, CD36, and CD74 were highly expressed in CD45^high^ CNS MPs (Figure 3B, lower row).

Since our proteomic data identified distinct phagocytic receptor profiles of LPS-activated and IL4-activated microglia, we compared the phagocytic characteristics of classically activated (LPS) and alternatively activated (IL4 or TGFβ/IL10) microglia. While LPS augmented both PE-microsphere and fAβ42 phagocytosis, IL4 was found only to augment fAβ42 phagocytosis while TGFβ/IL10 had no significant effect (Figure 3C) suggesting distinct phagocytic mechanisms for microsphere and fAβ42 phagocytosis. Next, we performed functional blockade experiments to target TLR2, PTPRJ/CD148, MARCO, MSR1, and CD36 in BV2 microglia (Figures 3D,E). We found that pre-incubation with anti-TLR2 mAb or anti-MARCO mAbs partially inhibited latex microsphere phagocytosis while mAbs against CD148, MSR1, and CD36 had no effect (Figure 3D). In contrast, fAβ42 phagocytosis was inhibited by anti-CD148, anti-CD36, and anti-MSR1 antibodies while anti-MARCO and anti-TLR2 mAbs had no effect (Figure 3E). These distinct patterns of phagocytosis inhibition suggest that fAβ phagocytosis involves CD148, CD36, and MSR receptors while microsphere phagocytosis involves TLR2 and MARCO receptors.

### Comparison of Phagocytic Capacity of CD11b^+^ CD45^low^, CD11b^+^ CD45^high^ CNS MPs and Peripheral Macrophages in Resting and Acute Neuroinflammatory States

**Figure 4:**
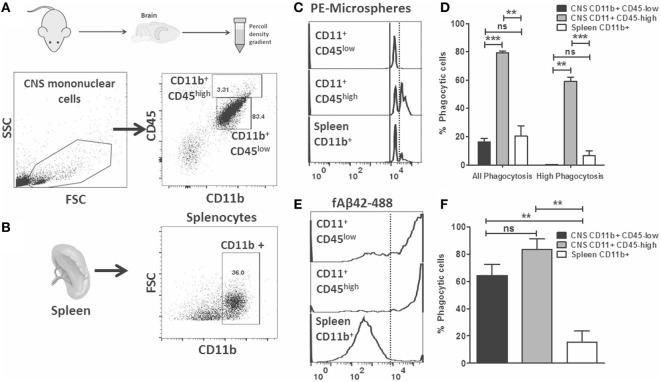
Flow cytometric microsphere and fAβ_42_ phagocytosis studies of acutely isolated CNS MPs from wild-type mice. **(A)** Outline of acute isolation of CNS MPs and typical scatter plot of live-gated CNS MPs based on CD11b and CD45 expression. Majority of cells are CD11b^+^ CD45^low^ while a small proportion of cells are CD11b^+^ CD45^high^. Contaminating lymphocytes (CD11b^neg^ CD45^high^) and other non-immune cells are also seen. **(B)** Typical scatter plot of acutely isolated live-gated and Ly6G negative splenocytes, further gated on CD11b^+^ status to enrich for splenic macrophages/monocytes. **(C,D)** Representative flow cytometric histograms showing distinct peaks of phycoerythrin (PE)-microsphere phagocytosis in both CD45^low^ and CD45^high^ CNS MPs as well as CD11b^+^ splenocytes. Analysis from five mice per group is shown in panel **(D)**. **(E,F)** Representative flow cytometric histograms showing fluorescent fAβ_42_ phagocytosis by CNS MPs and splenic CD11b^+^ cells. Analysis from five mice per group is shown in panel **(F)** (*p < 0.05, **p < 0.01, ***p < 0.005).

Flow cytometric phagocytosis assays allow rapid phenotyping of acutely isolated CNS MPs which are also more reflective of the complex microenvironment in the healthy and diseased brain (18, 19). We hypothesized that phagocytic properties of acutely isolated CNS MPs, including CD11b^+^CD45^low^ microglia and CD11b^+^ CD45^high^ CNS-infiltrating CNS MPs can be rapidly measured and compared using the above described phagocytosis assays. We performed *ex vivo* phagocytosis assays using acutely isolated CNS MPs (Figure 4A) as well as splenocytes (Figure 4B) from wild-type adult mice. Cells were incubated separately with fluorescent microspheres, fAβ42-488 (5 µM) or control for 30 min and then washed and then labeled for CD11b and CD45 (for CNS MPs) and CD11b (for splenocytes) and assayed by flow cytometry. As expected, microglia (CD11b^+^ CD45^low^) accounted for the majority of CD11b^+^ CNS MPs while CD11b^+^CD45^high^ CNS MPs represented 3% of CD11b^+^ immune cells in the brain. In the PE-microsphere phagocytosis assay, we observed distinct peaks of PE fluorescence in CD11b^+^ CD45^low^, CD11b^+^ CD45^high^ CNS MPs as well as in CD11b^+^ splenocytes (Figure 4C). However, CD11b^+^CD45^high^ CNS MPs had the highest phagocytic capacity for PE-microspheres (Figures 4C,D). In the fAβ42-488 phagocytosis assay, we observed very high phagocytic capacity in both CD45^low^ and CD45^high^ CNS MPs while splenic CD11b^+^ cells were poor phagocytosers of fAβ42 (Figures 4E,F). These results suggest fAβ42 phagocytic mechanisms may be induced selectively in the CNS. These data also support distinct mechanisms for PE-microsphere and fAβ42 phagocytosis.

**Figure 5:**
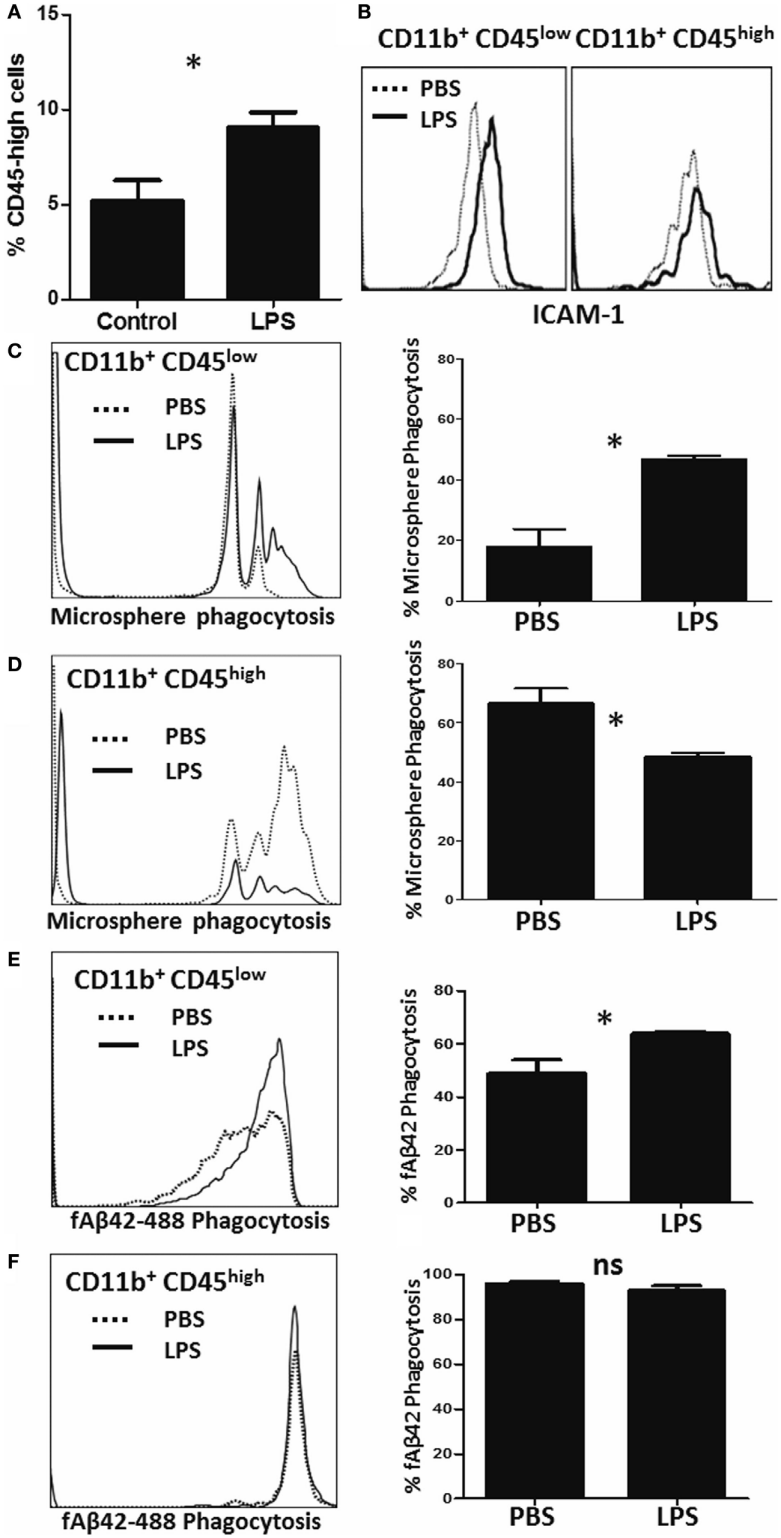
Characterization of microsphere and fAβ42 phagocytosis by CNS MPs in the lipopolysaccharide (LPS)-induced acute neuroinflammation mouse model. For these experiments, adult (3- to 4-months old) C57BL6/J mice were given four daily IP injections of LPS (10 µg) or PBS, after which CNS MPs were acutely isolated. **(A)** Comparison of proportions of CD11b^+^CD45^high^ CNS MPs in untreated and LPS-treated mice. **(B)** Comparison of pro-inflammatory marker ICAM-1 expression within CD45^high^ and CD45^low^ CNS MPs isolated from PBS and LPS-treated mice. **(C,D)** Comparison of phycoerythrin-microsphere phagocytosis in CD45^high^ and CD45^low^ CNS MPs from PBS and LPS-treated mice. **(E,F)** Comparison of fAβ42 phagocytosis in and CD45^low^ and CD45^high^ CNS MPs from PBS and LPS-treated mice. Data from six mice per treatment group are shown (*p < 0.05, **p < 0.01, ***p < 0.005).

To measure alterations in phagocytic capacities induced by neuroinflammation *in vivo*, we injected adult (3–4 months) WT mice with saline (PBS) or LPS (IP) for four consecutive days to induce a sickness response and separately measured PE-microsphere and fAβ42 phagocytosis *ex vivo* immediately after isolation from the brain (23). As expected, LPS increased the proportion of CD11b^+^CD45^high^ CNS MPs in the brain (Figure 5A) and increased the expression of pro-inflammatory marker ICAM-1 in both CD45^low^ and CD45^high^ CNS MPs (Figure 5B). In the PE-microsphere assay, LPS treatment significantly induced phagocytic capacity of CD45^low^ microglia (Figure 5C) and surprisingly decreased phagocytosis in the CD45^high^ CNS MPs (Figure 5D). In the fAβ42 phagocytosis assay, LPS augmented phagocytosis in CD45^low^ microglia (Figure 5E) while 90–100% of CD45^high^ CNS MPs phagocytosed fAβ_42_ regardless of LPS or PBS treatment (Figure 5F). Since CD45^low^ microglia represent the majority of CNS MPs isolated, we observed an overall augmentation of PE-microsphere and fAβ42 phagocytosis in LPS-treated mice compared to PBS-treated mice (data not shown).

### Characterization of Phagocytic Properties of CNS MPs in Aging Mice and in the 5xFAD Mouse Model of AD Pathology

**Figure 6:**
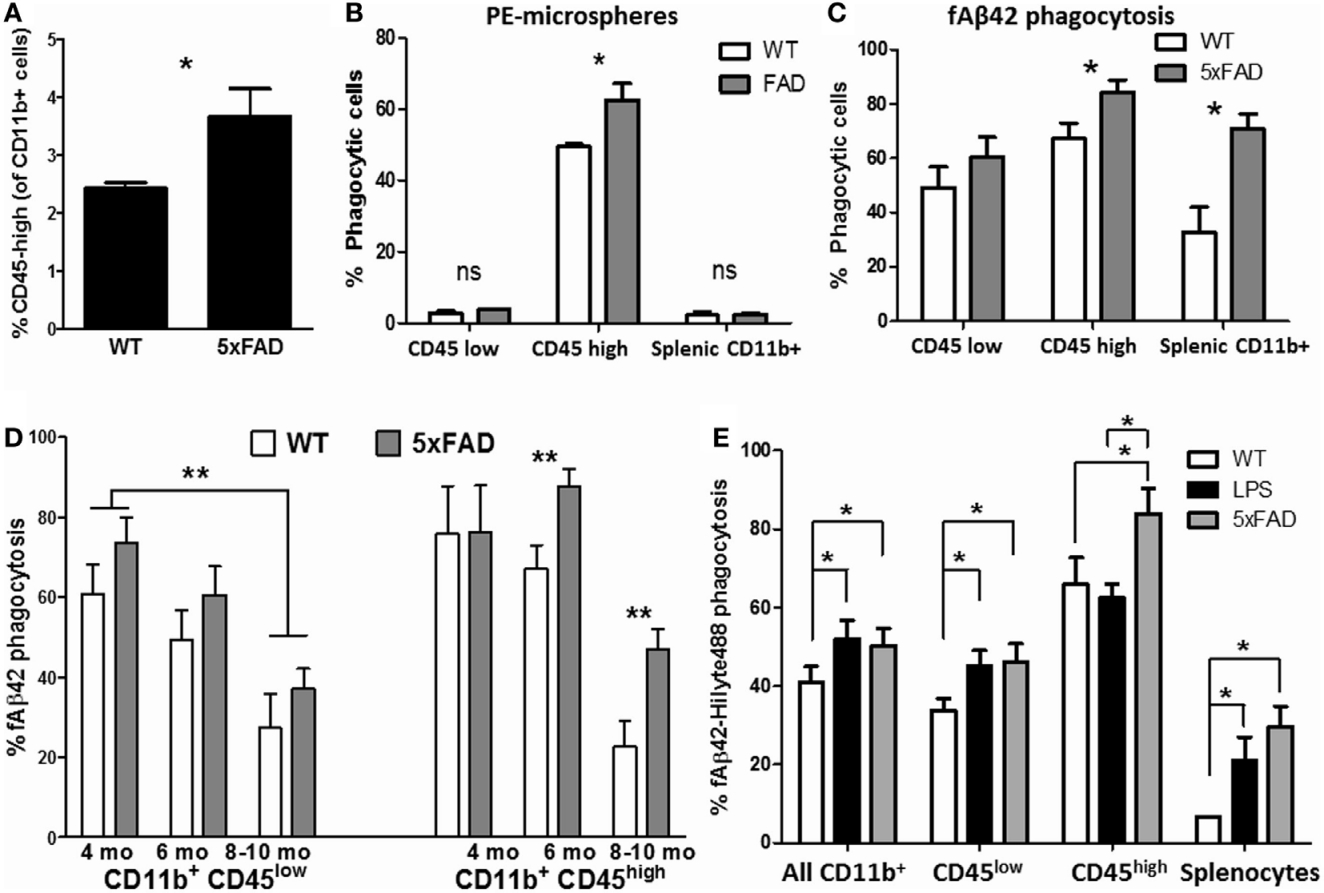
Characterization of phagocytic properties of CD45^low^ and CD45^high^ CNS MPs in the amyloidogenic 5xFAD mouse model of Alzheimer’s disease pathology. Acutely isolated CNS MPs and splenic CD11b^+^ cells from WT and 5xFAD mice at different ages were used for these flow cytometric studies. **(A)** Comparison of proportion of CD11b^+^ CD45^high^ CNS MPs in 6-month-old 5xFAD and WT mice (*n* = 9/group). **(B)** Comparison of phycoerythrin (PE)-microsphere phagocytosis in CD45^high^ and CD45^low^ CNS MPs from 6-month-old WT and 5xFAD mice (*n* = 6/group). **(C)** Comparison of fAβ42 phagocytosis in CD45^high^ and CD45^low^ CNS MPs from 6-month-old WT and 5xFAD mice (*n* = 6/group). **(D)** Comparison of fAβ42 phagocytosis in 5xFAD and WT CNS MPs at 3–4, 5–6, and 8–10 months of age (*n* = 3 mice per group). **(E)** Comparison of fAβ_42_ phagocytosis in CD45^high^ and CD45^low^ CNS MPs and Splenic CD11b^+^ cells from untreated WT, LPS-treated WT and age-matched 5xFAD mice (age 6 months, *n* = 3 mice per group); *p < 0.05, **p < 0.01, ***p < 0.005.

Aβ phagocytosis and clearance is a protective microglial response in the brain that becomes progressively dysregulated in states of chronic neuroinflammation as seen in AD (58). Therefore, we studied phagocytic profiles of acutely isolated CNS MPs in the 5xFAD mouse model of AD pathology in which rapid accumulation of Aβ plaques begins at 3 months of age followed by neurodegeneration and neurobehavioral deficits by 8–10 months of age (59). 5xFAD mice consistently demonstrated a higher proportion of CD11b^+^ CD45^high^ CNS MPs as compared to WT age/sex-matched mice (Figure 6A). At age 6–7 months, CD45^high^ CNS MPs from 5xFAD mice were found to be more efficient phagocytosers of PE-microspheres (Figure 6B) as well as of fAβ42 (Figure 6C, *p* = 0.02). CD11b^+^ CD45^low^ microglia from WT and 5xFAD mice exhibited similar PE-microsphere and fAβ42 phagocytic efficiency while splenic CD11b^+^ macrophages from 5xFAD mice were more efficient at phagocytosing fAβ42 compared to splenic CD11b^+^ cells from WT mice (Figures 6B,C). To study the effect of aging and progressive Aβ accumulation on fAβ42 phagocytosis, we assessed CD11b^+^ CNS MPs from WT and 5xFAD mice at 4, 6, and 8–10 months of age (Figure 6D). In WT mice, both CD45^low^ and CD45^high^ CNS MPs demonstrated an age-dependent reduction in fAβ42 phagocytic capacity. In comparison, CD45^high^ CNS MPs from 5xFAD mice showed persistently elevated phagocytic capacity for fAβ42 at 8 and 10 months of age. In contrast to fAβ42 phagocytosis, we found that aging, irrespective of WT or 5xFAD background, resulted in increased PE-microsphere phagocytic capacity of CD11b^+^ CNS MPs, although this effect of aging was more pronounced in 5xFAD mice (Figure S2 in Supplementary Material).

In an independent experiment, we directly compared fAβ42 phagocytic capacity of CNS MPs acutely isolated from 6-month-old untreated WT, LPS-treated WT and 5xFAD mice (Figure 6E). Overall, CD11b^+^ CNS MPs including CD11b^+^ CD45^low^ microglia from LPS-treated and 5xFAD mice showed higher phagocytic capacity as compared to WT mice. Within the CD11b^+^ CD45^high^ subgroup, CNS MPs from 5xFAD mice showed enhanced phagocytic activity compared to LPS-treated and untreated WT mice. Overall, these data support divergent regulation of phagocytosis within CD45^low^ and CD45^high^ CNS MPs in acute neuroinflammatory and chronic neurodegenerative disease states. The results also suggest that microsphere and fAβ_42_ phagocytosis involve distinct phagocytic mechanisms that are differentially regulated during aging and disease.

### CD11b^+^CD45^high^ CNS MPs in Neurodegenerative Disease Do Not Express Peripheral Myeloid Marker Ly6c but Express High CD44 Levels

**Figure 7:**
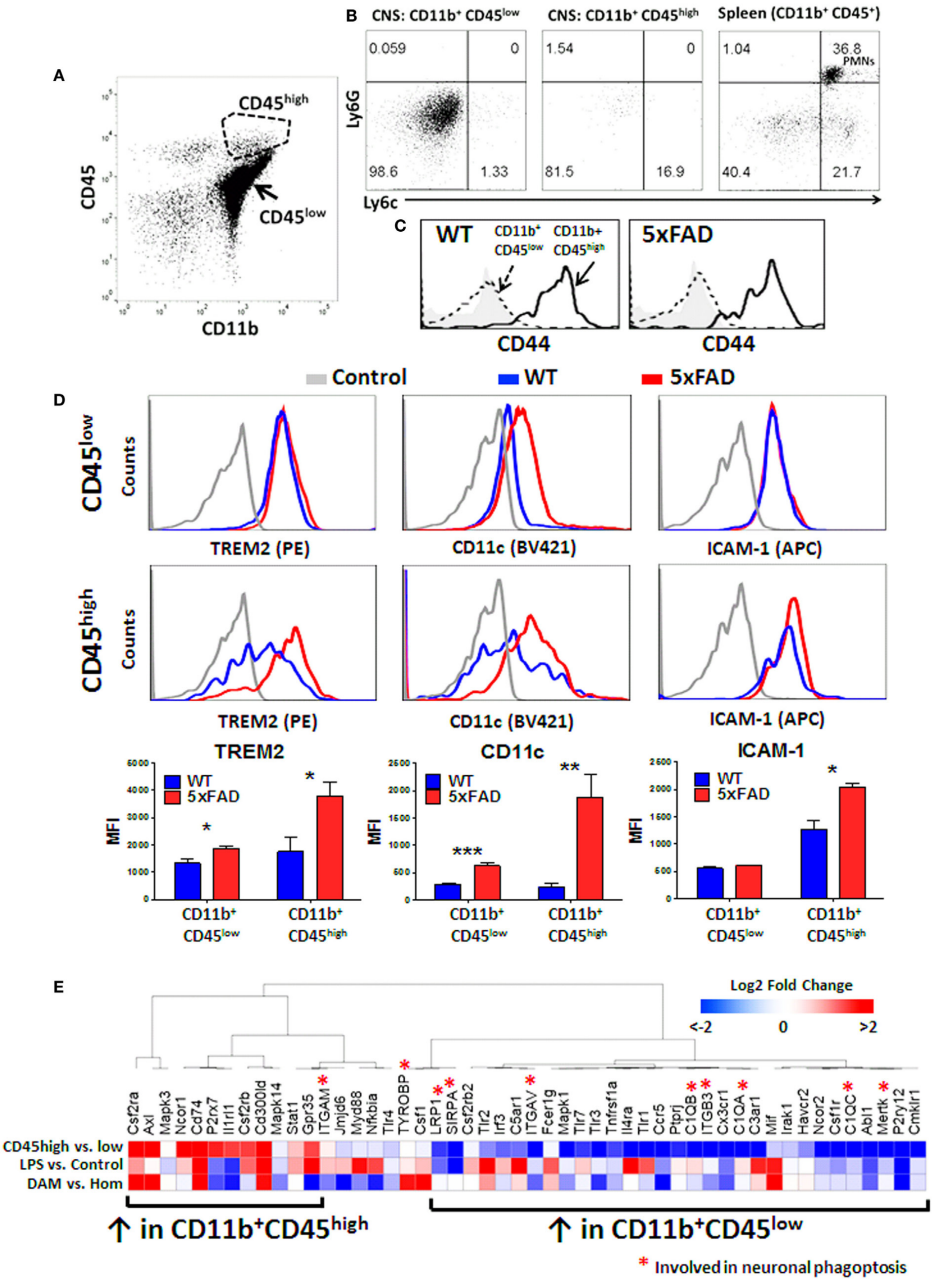
CD45^high^ CNS MPs do not express peripheral origin marker Ly6c but highly express CD44 and phagocytic genes TREM2, CD11c, and disease-associated microglia (DAM)-signature genes in 5xFAD mice. **(A)** Scatter plot demonstrating the CD45^high^ and CD45^low^ subsets of CNS MPs in 8-month-old 5xFAD mice. **(B)** Expression of Ly6c (marker of peripheral origin) and Ly6G (neutrophil marker) in CD11b^+^CD45^low^, CD11b^+^ CD45^high^ CNS MPs and CD11b^+^CD45^+^ splenocytes. **(C)** Comparison of CD44 expression in CD11b^+^CD45^low^ and CD11b^+^ CD45^high^ CNS MPs in WT and 5xFAD mice. **(D)** Comparison of cell surface expression of phagocytic receptor TREM2, CD11c and activation marker ICAM-1 on CD45^high^ and CD45^low^ CD11b^+^ CNS MPs. Quantification of data from three WT and three 5xFAD mice (ages 6–8 months) is shown below. **(E)** A comparative transcriptomic analysis of phagocytic receptor genes identified by RNAseq in CD11b^+^ CD45^low^ and CD45^high^ CNS MPs, LPS-activated CD45^low^ microglia, and DAM and homeostatic microglia in WT and 5xFAD mice. Color scheme used indicates magnitude of log2-fold difference as shown in the scale bar.

The origin of CD11b^+^CD45^high^ CNS MPs is unclear, but in acutely isolated CNS MPs, minor contamination by CD11b^+^ CD45^+^ blood monocytes (Ly6c^+^ Ly6G^−^), CD11b^+^ CD45^+^ neutrophils (Ly6c^+^Ly6G^+^) and lymphocytes (CD11b^−^ CD45^+^) is possible despite sufficient cardiac perfusion. To confirm that CD11b^+^ CD45^high^ CNS MPs were indeed CNS-resident cells rather than blood contaminants, we performed flow cytometric studies of acutely isolated CNS MPs from adult WT mice and included markers of peripherally derived CNS-infiltrating monocytes/macrophages (Ly6c) and neutrophils (Ly6G) along with CD11b and CD45 in the panel (Figures 7A,B). Unlike CD11b^+^ splenocytes that comprised of Ly6G^−^ Ly6c^high^ monocytes and Ly6G^+^Ly6c^+^ neutrophils, both CD45^low^ and CD45^high^ CNS MPs expressed minimal Ly6c and no Ly6G. We also found that CD44, recently suggested as a marker of peripherally derived CNS-infiltrating immune cells in mice (60), was highly expressed by CD11b^+^CD45^high^ CNS MPs but not by CD45^low^ CNS MPs in both WT and 5xFAD mice (Figure 7C). These data suggest that CD45^high^ cells may originate as a subpopulation of CD45^low^ microglia that strongly upregulate CD45 and CD44 although it is still possible that peripheral Ly6c^high^ and CD44^high^ monocytes/macrophages downregulate Ly6c but not CD44 after entering the brain (61).

### Triggered Receptor Expressed on Myeloid Cells 2 (TREM2) Phagocytic Receptor and DAM-Signature Genes Are Highly Upregulated by CD11b^+^CD45^high^ CNS MPs

TREM2 is a phagocytic receptor expressed by macrophages and microglia that is involved in clearance of Aβ and has been directly implicated in AD pathogenesis (9, 13, 62, 63). In AD mouse models, deletion of TREM2 accelerates Aβ accumulation and neuronal loss while overexpression ameliorates neuropathology in mouse AD models suggesting a protective Aβ-clearing role for TREM2 in AD (9, 13, 64). TREM2 gene expression is also upregulated by a subpopulation of DAM, which progressively accumulate in the aging 5xFAD mouse brain with possible Aβ clearing and protective functions (37). TREM2 also serves as a checkpoint for the emergence of DAM (37). At the transcriptomic level, TREM2 is expressed at 30-fold higher levels in CD45^high^ CNS MPs and over 500-fold higher levels in CD45^low^ CNS MPs as compared to the whole brain in wild-type mice, although following *in vivo* LPS exposure, TREM2 mRNA levels in CD45^low^ microglia are more comparable to CD45^high^ CNS MPs (12). Since our data suggest that CD45^high^ CNS MPs have higher phagocytic capacity for fAβ especially in the 5xFAD brain, we hypothesized that TREM2 expression is also increased in CD11b^+^CD45^high^ CNS MPs, and assessed expression of the cell surface TREM2 in WT and 5xFAD mice by flow cytometry. In 6-month-old mice, we found that TREM2 expression in CNS MPs was significantly higher than splenic macrophages in WT and 5xFAD mice (Figure S3 in Supplementary Material). In WT mice, TREM2 expression was similar in CD45^low^ and CD45^high^ CNS MPs whereas in 5xFAD mice, CD45^high^ CNS MPs strongly upregulated TREM2 expression while CD45^low^ CNS MPs showed a marginal yet significant increase in TREM2 expression (Figure 7D). Increased TREM2 expression in CD45^high^ cells also paralleled increased expression of DAM marker CD11c as well as activation marker ICAM-1 (Figure 7D).

We next performed an integrative analysis of genes involved in phagocytosis or phagoptosis that are expressed by CD11b^+^CD45^high^ and CD45^low^ CNS MPs using existing population-level RNAseq data from adult WT mice (untreated and LPS-treated) and single-cell RNAseq data from WT and 5xFAD mouse CD45^+^ CNS MPs (12, 37). 5,434 genes were identified across both RNAseq datasets with no missing values and among these, we identified 51 phagocytic or phagoptotic genes (Table S3 in Supplementary Material). An unsupervised hierarchical clustering analysis of relative expression data broadly segregated the CD45^low^ and CD45^high^ CNS MP genes (Figure 7E; Figure S4 in Supplementary Material). LPS-upregulated genes clustered with genes that were also highly expressed by CD45^high^ CNS MPs. DAM-signature genes, including CSF2RA, Axl, Mif, CD74, and CSF1, were also highly expressed in CD45^high^ population CNS MPs. Within the list of 51 genes used for this analysis, 10 genes are implicated in live neuronal phagocytosis (or phagoptosis) which in neurodegenerative diseases, can be detrimental by promoting death of viable neurons that express distress signals (65). Eight of these 10 phagoptosis genes were highly expressed in CD45^low^ microglia. These observations highlight unexpected similarities in phagocytic gene expression between DAM, pro-inflammatory activated microglia and CD11b^+^CD45^high^ CNS MPs. Overall, these data show that CD11b^+^CD45^high^ CNS MPs have high affinity for fAβ42, express high TREM2 and CD11c levels and also highly express several phagocytic genes that are characteristic of DAM cells that are observed in AD, suggesting their protective Aβ-clearing role. Unlike CD45^high^ CNS MPs, CD45^low^ microglia express phagoptosis genes that can be potentially detrimental in neurodegenerative disease.

## Discussion

Phagocytic clearance of debris, infectious agents, and protein aggregates are key functions of phagocytes, which within the brain are primarily performed by CD11b^+^ CD45^low^ microglia and a small proportion of CD45^high^ CNS MPs which may represent either CNS-infiltrating peripheral macrophages or activated microglia that upregulate CD45. In the healthy adult brain and in chronic neurodegenerative disease states, CD45^high^ CNS MPs express low levels of Ly6c and CCR2 supporting their origin from microglia although it is still possible that peripherally derived macrophages downregulate Ly6c and CCR2 after residing in the CNS (66). Due to the low prevalence of CD45^high^ CNS MPs in the brain, these cells are often excluded from microglial transcriptomic and functional studies (11, 67). In a RNAseq study of mouse CNS MPs by Bennett et al., differences in transcriptomic signatures of CD45^low^ and CD45^high^ cells were identified, suggesting enrichment of LXR/RXR-mediated anti-inflammatory and cholesterol efflux pathways in CD45^low^ microglia while CD45^high^ CNS MPs showed enrichment for genes regulating leukocyte trafficking, integrin signaling, and activation of coagulation (12). The presence of CD45^+^ TREM2^+^ CNS MPs around Aβ plaques in AD mouse models suggest a pro-phagocytic and Aβ-clearing role for CD45^high^ cells (68), but direct functional characterization of phagocytic properties of CD45^high^ CNS MPs, in contrast to CD45^low^ CNS MPs, has not been performed.

**Figure 8:**
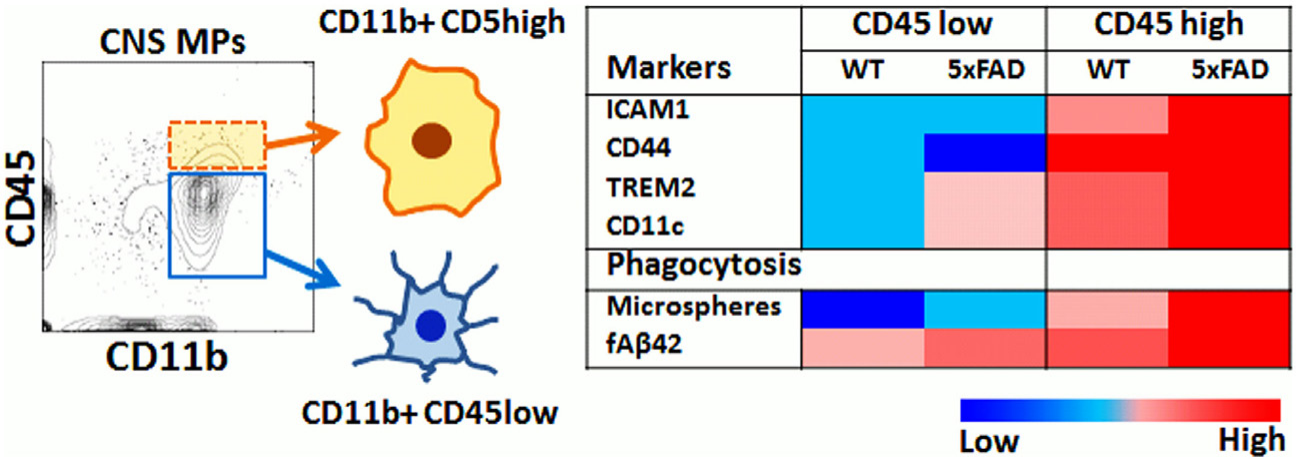
Summary of flow cytometric and phagocytic characteristics of CD45^high^ and CD45^low^ CNS MPs.

In this study, we have applied rapid flow cytometric assays of phagocytosis to directly compare the phagocytic properties of CD11b^+^CD45^high^ and CD11b^+^CD45^low^ acutely isolated CNS MPs in homeostatic, acute, and chronic neuroinflammatory conditions and a summary of our key findings is provided in Figure 8. Specifically, CD11b^+^ CD45^high^ CNS MPs are more efficient bulk-phase phagocytosers of macroparticles (such as latex microspheres) as compared to CD11b^+^ CD45^low^ microglia and splenic macrophages. On the other hand, most CNS MPs (regardless of CD45^high^ or CD45^low^ expression) are avid phagocytosers of fAβ42 as compared to splenic macrophages suggesting that the CNS environment specifically induces Aβ-clearing mechanisms in CNS MPs. In a model of acute neuroinflammation induced by systemic LPS, we observed augmented microsphere and fAβ42 phagocytosis in the CD45^low^ microglial population and an unexpected reduction in phagocytic capacity of CD45^high^ CNS MPs for microspheres without alteration in the high fAβ42-phagocytosing capacity of CD45^high^ CNS MPs. In the 5xFAD model of chronic neurodegeneration and Aβ accumulation, CD45^low^ CNS MPs showed augmented microsphere and fAβ42 phagocytic capacity in a similar manner seen in LPS-induced acute neuroinflammation. However, CD45^high^ CNS MPs from 5xFAD mice showed higher fAβ_42_ phagocytosis as compared to WT and LPS-treated mice, suggesting a unique affinity for fAβ42 in this population. Aging resulted in increased microsphere phagocytosis while fAβ42 phagocytic capacity decreased in CNS MPs. However, CD45^high^ CNS MPs in 5xFAD mice retained their high fAβ42 phagocytic capacity. In 5xFAD mice, CD45^high^ CNS MPs also highly upregulated the phagocytic receptor TREM2 as compared to CD45^low^ microglia, an observation that agrees with Jay et al. who also found that 5xFAD CD45^high^ CNS MPs selectively upregulated TREM2 expression in an age-dependent manner (69). By integrating existing RNAseq datasets from CD45^high^ and CD45^low^ CNS MPs (12), with recent single-cell RNAseq data (37), we also observed that phagocytic genes highly expressed by CD45^high^ CNS MPs show a surprising clustering with DAM-signature phagocytic genes including CD300ld, CSF1, MIF, Axl, and CSF2ra. Although the functional relevance of DAM to AD pathogenesis has not been directly verified, their transcriptomic signatures, accumulation around Aβ plaques, and increased amounts of intracellular Aβ support phagocytic and protective roles for DAM in AD. We conclude that CD45^high^ CNS MPs may have high Aβ clearing and protective properties as compared to CD45^low^ microglia in AD. Whether this augmented phagocytic capacity of CD45^high^ cells in advanced stages of AD pathology translates to more effective Aβ clearance, is unclear. Since the majority of microglia (CD45^low^) show decreased Aβ phagocytosing ability with aging and progression of Aβ accumulation, it is possible that chronic dysregulation of Aβ phagocytosis in microglia is a deleterious response that is partly counterbalanced by Aβ clearing CD45^high^ CNS MPs, especially in early stages of disease. Since CD45^high^ cells represent <10% of all acutely isolated CNS MPs even in the aged 5xFAD brain, it is likely that any beneficial effects of TREM2^++^ CD45^high^ cells are overshadowed by inability of CD45^low^ CNS MPs to clear Aβ in advanced stages of AD.

With regard to the origin of CD45^high^ CNS MPs in the WT and 5xFAD brain, the absence of Ly6c expression in majority of CD45^high^ cells in our studies argue against a peripheral monocyte origin for these cells. This contradicts conclusions by Jay et al. who showed that Ly6c was expressed by CD45^+^ cells that surround Aβ plaques in 5xFAD mice (69). A possible explanation for this disagreement is that we measured cell surface Ly6c and CD45 by flow cytometry while Jay et al. detected Ly6c and CD45 by immunohistochemistry which detects both intracellular and cell surface proteins. Ly6c gene expression is also increased nearly 100-fold by CD45^low^ microglia following LPS activation *in vivo* (one-fourth the expression of CD45^high^ CNS MPs) as measured by RNAseq (12). Depending on the limit of detection of anti-Ly6c antibodies used in immunohistochemistry, it is very likely that upregulated Ly6c expression by activated microglia, rather than Ly6c positivity in peripheral monocytes/macrophages, was detected. Therefore, we suggest that the co-expression of TREM2 along with CD45 and Ly6c in plaque-associated CNS MPs as detected by immunohistochemistry does not prove that TREM2^+^CD45^+^ CNS MPs are of peripheral monocyte origin. A more recent CyTOF study of CNS MPs from 8- to 10-week mice found that CD44 exclusively identified cells of peripheral origin (60). We found that CD44 in 4-month-old mice was highly expressed by all CD11b^+^CD45^high^ but not CD11b^+^CD45^low^ CNS MPs in WT and 5xFAD mice, suggesting that CD11b^+^CD45^high^ CNS MPs may be of peripheral origin but it still remains possible that CD44 expression is upregulated by microglia with aging and in inflammatory states. Our contradictory findings with Ly6c and CD44 highlight the uncertainty in the field regarding origin of CD11b^+^ CD45^high^ CNS MPs.

In addition to highlighting the differences between CD45^low^ and CD45^high^ CNS MPs, we also observed that splenocytes from 5XFAD and LPS-treated mice had an unexpectedly higher capacity to phagocytose fAβ42 compared to untreated wild-type mice, while splenocytes were poor phagocytosers of PE-microspheres. Although we did not perform additional immunophenotyping of CD11b^+^Ly6G^−^ splenocytes to discern subtypes of monocytes, macrophages, or dendritic cells, this intriguing observation supports the idea that peripheral macrophages, like CD45^high^ CNS MPs in the brain, may specifically upregulate the phagocytic machinery necessary to phagocytose Aβ. Furthermore, the observed priming of splenic macrophages for fAβ in 5xFAD mice also supports the existence of a “brain-spleen immune axis,” which may involve cholinergic pathways (70, 71). Since the adaptive immune system may limit AD pathogenesis by modulating microglia in AD mouse models (72), the relevance of higher fAβ42 phagocytosis exhibited by peripheral macrophages to CNS pathology and Aβ clearance needs to be further investigated.

We also provide mechanistic insights into receptors that regulate microsphere and fAβ42 phagocytosis in murine microglia. While bulk-phase phagocytosis of microspheres involves TLR2 and MARCO receptors, fAβ phagocytosis requires CD148, CD36, and MSR1 receptors. While LPS, a pro-inflammatory stimulus, is known to upregulate MARCO, TLR2, and CD148 expression in microglia, we found that IL4-mediated M2-like activation results in CD36 upregulation. TLR2-dependent phagocytosis under acute inflammatory conditions is associated with pro-inflammatory cytokine release, cell killing, neurotoxicity, and neuronal phagoptosis of live neurons while CD36 is implicated in phagocytosis of apoptotic cells resulting in anti-inflammatory and protective responses (11, 45, 46, 73–75). TLR2 is highly specific to CD45^low^ microglia while CD36 and MSR1 are highly specific to the CD45^high^ CNS MP population. These suggest that the phagocytic receptor profile expressed by CNS MPs is tightly linked to the inflammatory stimulus that the cell is exposed to in the microenvironment. Furthermore, engagement of these distinct phagocytic receptors may influence downstream pro- or anti-inflammatory effector functions, including cytokine production, reactive oxygen species production, and neurotoxicity. Since CD45^low^ microglia express majority of phagoptosis genes including TLR2 (65), while CD45^high^ CNS MPs more highly express DAM-specific and fAβ phagocytic receptors, we propose that the distinct phagocytic receptor profiles of CD45^low^ and CD45^high^ CNS MPs may reflect an overall pro-phagocytic and Aβ-clearing and potentially neuroprotective function for CD45^high^ CNS MPs in neurodegenerative disease and aging. Finally, our results also highlight significant differences in phagocytic receptor expression and regulation between primary murine microglia and BV2 microglia. Of several phagocytic proteins upregulated by LPS-treated BV2 microglia, only MARCO and TLR2 showed congruent changes in primary microglia. The BV2 cell line is also not an adult microglial cell line, further emphasizing the need to validate findings from BV2 cells or from *in vitro* cultured primary microglia in acutely isolated adult microglia.

Some limitations of our studies need to be highlighted. We have primarily focused on bulk-phase and fAβ phagocytosis although these flow cytometric assays need to be adapted to study phagocytosis and endocytosis of oxidized-LDL particles, infectious agents, apoptotic cells, healthy neurons, and opsonized particles, all of which involve distinct phagocytic mechanisms implicated in neurological diseases (5). We have also limited our studies in neurodegenerative disease to the 5xFAD model that mostly recapitulates Aβ accumulation and neurodegeneration without significant tau pathology, a significant component of human AD pathology (59, 76). The 5xFAD model also demonstrates very rapid Aβ accumulation unlike other AD models. Future characterization of CNS MPs from slowly progressive amyloidopathy and tauopathy models of neurodegeneration as well as of acutely isolated postmortem human CNS MPs will help clarify the clinical relevance of our findings. Finally, while we have used existing transcriptomic data from CD45^high^ and CD45^low^ CNS MPs from adult WT mice in our analyses, global transcriptomic or proteomic profiles of CD45^high^ and CD45^low^ CNS MPs in the AD disease models have not been elucidated.

In summary, we have comprehensively validated two flow cytometric approaches to study phagocytic responses that can be readily applied to the study of acutely isolated microglia. We have highlighted novel differences in phagocytic capacities in CD45^low^ microglia and CD45^high^ CNS MPs under homeostatic, acute neuroinflammatory and neurodegenerative disease states. We demonstrate that CD45^high^ CNS MPs are more efficient bulk-phase phagocytosers as compared to CD45^low^ microglia and express gene profiles that resemble activated microglia as well as some characteristics specific to the recently identified DAM population. In advanced neurodegenerative disease states and with aging, a global suppression in Aβ phagocytic efficiency is observed although CD45^high^ CNS MPs still remain relatively highly phagocytic. CD45^high^ cells also express higher TREM2 as compared to CD45^low^ cells, suggesting a protective effect of these cells. As compared to WT CD45^high^ cells, CD45^high^ cells from 5xFAD mice also have higher affinity for Aβ phagocytosis. Identifying ways to augment the numbers of CD45^high^ CNS MPs and promoting their Aβ phagocytic capacity may provide therapeutic opportunities in neurodegenerative disease.

## Ethics Statement

Institutional Animal Care and Use Committee approval was obtained prior to *in vivo* work and all work was performed in strict accordance with the Guide for the Care and Use of Laboratory Animals of the National Institutes of Health.

## Author Contributions

SR and SAR have conceptualized and executed all experiments, analyzed data, written this manuscript, and equally contributed to the work. RB and NL have performed experiments, analyzed data and critically reviewed and revised the manuscript. ED and DD performed proteomic studies and analyzed data. NS, JL, and AL have reviewed and discussed the results and have edited the manuscript.

## Conflict of Interest Statement

The authors declare that the research was conducted in the absence of any commercial or financial relationships that could be construed as a potential conflict of interest.
