# Supplementary material for: Differential Phagocytic Properties of CD45low Microglia and CD45high Brain Mononuclear Phagocytes—Activation and Age-Related Effects
Source: Front Immunol. 2018 Mar 2;9:405. doi: 10.3389/fimmu.2018.00405 (PMC5840283; doi:10.3389/fimmu.2018.00405)
Supplement: Supplementary file 2 [file data_sheet_2.DOCX]

**Differential phagocytic properties of CD45^low^ microglia and CD45^high^ brain mononuclear phagocytes – activation and age-related effects**

**Supplemental Information**

Authors:

Srikant Rangaraju,^#^ *Corresponding Author*, Department of Neurology, Emory University. Atlanta, GA 30322. Email: [srangar@emory.edu](mailto:srangar@emory.edu)

Syed Ali Raza,^#^ Department of Neurology, 615 Michael Street, Suite 525, Emory University, Atlanta, GA 30322. Email: [saraza2@emory.edu](mailto:saraza2@emory.edu)

Noel Xiang’An Li, Department of Chemistry, 1515 Dickey Dr. NE, Emory University, Atlanta, GA 30322, Email: [noel.li@emory.edu](mailto:noel.li@emory.edu)

Ranjita Betarbet, Department of Neurology, 615 Michael Street, Suite 525, Emory University, Atlanta, GA 30322. Email: [rbetarb@emory.edu](mailto:rbetarb@emory.edu)

Eric B. Dammer, Department of Neurology, 615 Michael Street, Suite 525, Emory University, Atlanta, GA 30322. Email: [edammer@emory.edu](mailto:edammer@emory.edu)

Duc Duong, Department of Biochemistry, 615 Michael Street, Suite 525, Emory University, Atlanta, GA 30322, Email: [dduong@emory.edu](mailto:dduong@emory.edu)

James J. Lah, Department of Neurology, 615 Michael Street, Suite 525, Emory University, Atlanta, GA 30322. Email: [jlah@emory.edu](mailto:jlah@emory.edu)

Nicholas T. Seyfried, Department of Biochemistry, 615 Michael Street, Suite 525, Emory University, Atlanta, GA 30322, Email: [nseyfri@emory.edu](mailto:nseyfri@emory.edu)

Allan I. Levey, Department of Neurology, 615 Michael Street, Suite 525, Emory University, Atlanta, GA 30322. Email: [alevey@emory.edu](mailto:alevey@emory.edu)

# Co-first authors


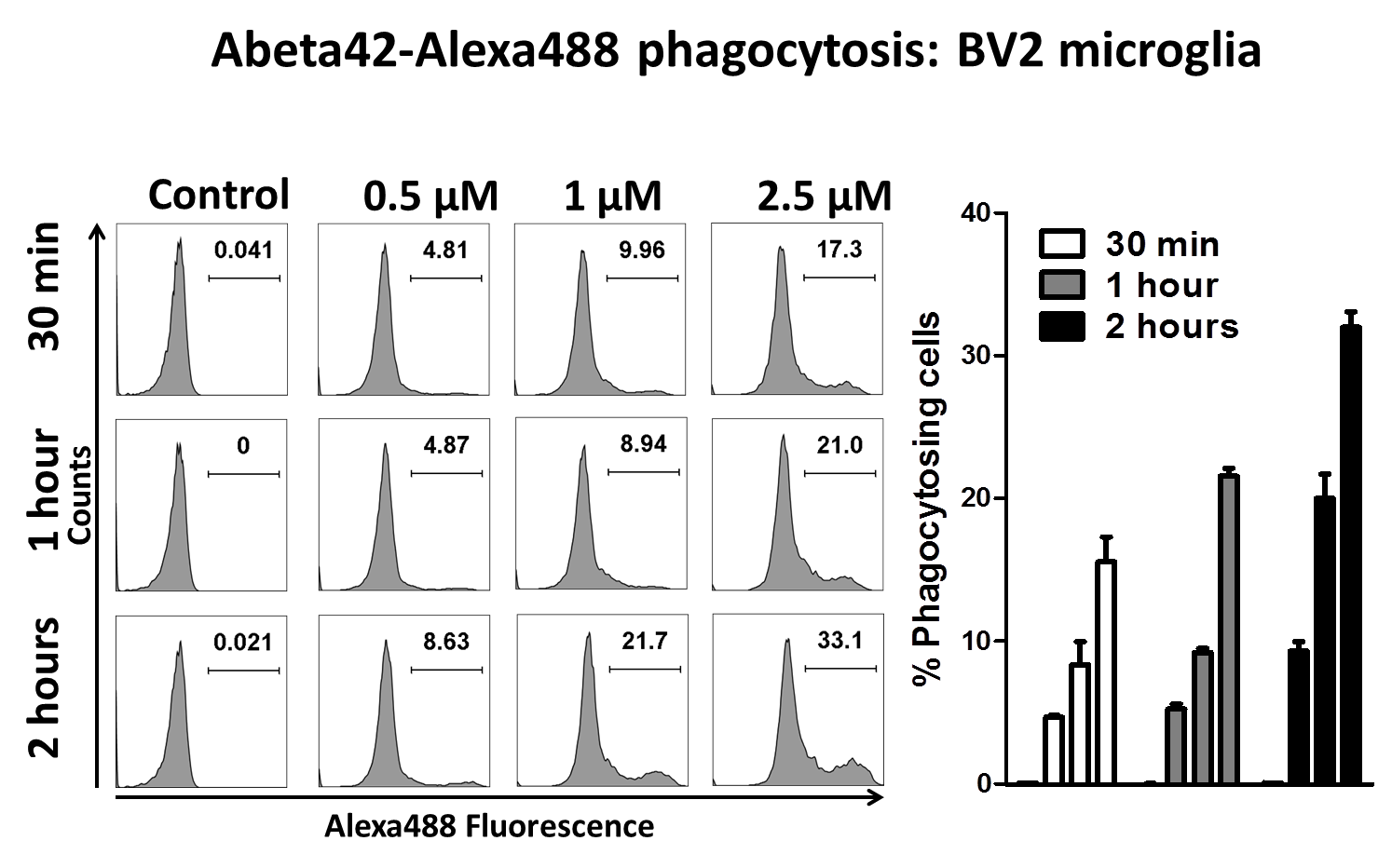
**Supplemental Figure 1. Optimization of fAβ42-Hilyte488 phagocytosis assay.** BV2 cells were treated with varying concentrations of fAβ42-Hilyte488 for 30 min, 1hr or 2 hours after which flow-cytometric analysis was performed. N=3 independent experiments per time point. Proportion of phagocytic cells are shown in each panel.


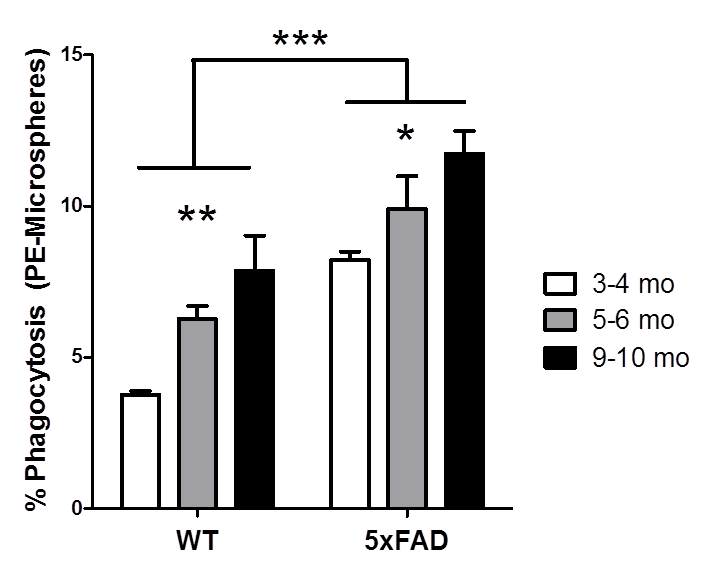


**Supplemental Figure 2. Aging results in augmentation of PE-microsphere phagocytic capacity in CNS MPs.** Acutely isolated CNS MPs from 6 mo WT and 5xFAD were used for these assays. PE microsphere phagocytic capacity was measured in all CD11b+ myeloid cells in the brain and group-wise and pair-wise comparisons were performed (n=3 mice/group, *p<0.05, **p<0.01, ***p<0.005).


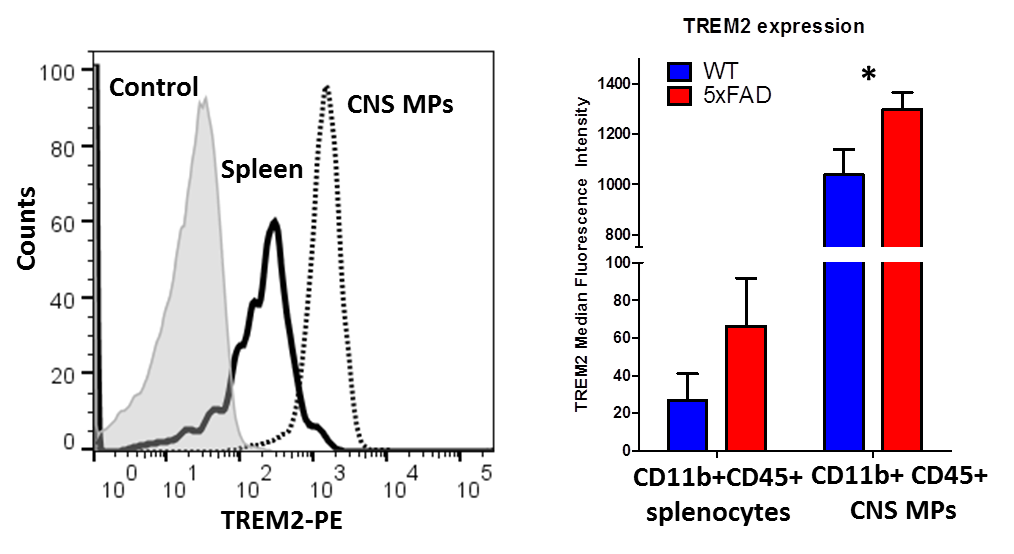


**Supplemental Figure 3.** Flow cytometric measurement of cell surfaceTREM2 expression by CD11b+CD45+ CNS MPs and splenic macrophages/monocytes. Acutely isolated CNS MPs and splenocytes from three adult (4-6 mo) WT mice were used for this experiment (*p<0.05). Median fluorescence intensity was compared across all groups after subtraction of background fluorescence.

**
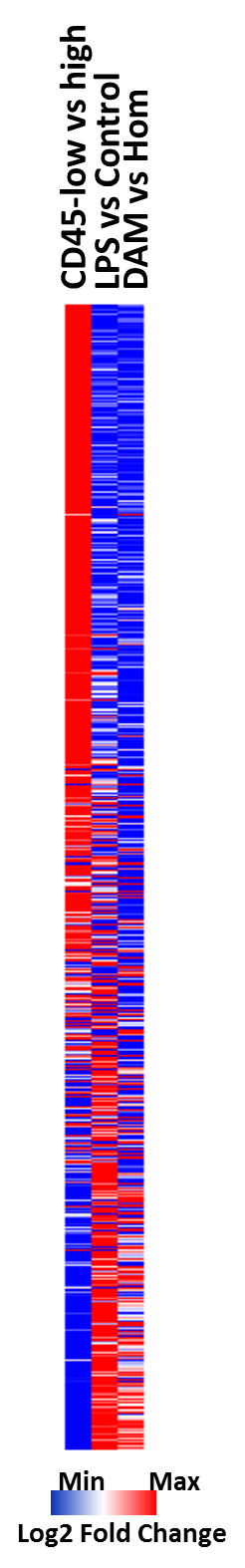
Supplemental Figure 4. Transcriptomic profiles of CD11b^+^CD45^high^ CNS MPs more closely resemble LPS-activated and DAM profiles.** Relative expression data from two publicly available RNAseq datasets (Dataset 1: RNAseq of CD11b^+^CD45^low^ and CD11b^+^CD45^high^ CNS MPs from adult WT mice, and RNAseq of CD45^low^ CNS MPs from WT and LPS-treated WT mice; Dataset 2: Single cell RNAseq of CD45+ immune cells from WT and 5xFAD mice) were log2 transformed, followed by hierarchical cluster analysis and a heat map was generated using Morpheus (Broad Institute). All genes (n=5,434) that were present in both datasets were used for this analysis.
